# Formation of 53BP1 foci and ATM activation under oxidative stress is facilitated by RNA:DNA hybrids and loss of ATM-53BP1 expression promotes photoreceptor cell survival in mice

**DOI:** 10.12688/f1000research.15579.1

**Published:** 2018-08-10

**Authors:** Vaibhav Bhatia, Lourdes Valdés-Sánchez, Daniel Rodriguez-Martinez, Shom Shankar Bhattacharya

**Affiliations:** 1 CABIMER (Centro Andaluz de Biología Molecular y Medicina Regenerativa), (FPS) Fundacion Progreso y Salud, Sevilla, Andalucia, 41092, Spain

**Keywords:** RNA:DNA-hybrids, ATM, 53BP1, Genome instability, Oxidative stress, DNA repair, Photoreceptor cell death, Retinal degeneration, Retinitis pigmentosa

## Abstract

**Background:** Photoreceptors, light-sensing neurons in retina, are central to vision. Photoreceptor cell death (PCD) is observed in most inherited and acquired retinal dystrophies. But the underlying molecular mechanism of PCD is unclear. Photoreceptors are sturdy neurons that survive high oxidative and phototoxic stress, which are known threats to genome stability. Unexpectedly, DNA damage response in mice photoreceptors is compromised; mainly due to loss of crucial DNA repair proteins, ATM and 53BP1. We tried to understand the molecular function of ATM and 53BP1 in response to oxidative stress and how suppression of DNA repair response in mice retina affect photoreceptor cell survival.

**Methods: **We use the state of art cell biology methods and structure-function analysis of mice retina. RNA:DNA hybrids (S9.6 antibody and Hybrid-binding domain of RNaseH1) and DNA repair foci (gH2AX and 53BP1) are quantified by confocal microscopy, in retinal sections and cultured cell lines. Oxidative stress, DNA double strand break, RNaseH1 expression and small-molecule kinase-inhibitors were used to understand the role of ATM and RNA:DNA hybrids in DNA repair. Lastly, retinal structure and function of ATM deficient mice, in Retinal degeneration 1 (Pde6brd1) background, is studied using Immunohistochemistry and Electroretinography.

**Results:** Our work has three novel findings: firstly, both human and mice photoreceptor cells specifically accumulate RNA:DNA hybrids, a structure formed by re-hybridization of nascent RNA with template DNA during transcription. Secondly, RNA:DNA-hybrids promote ataxia-telangiectasia mutated (ATM) activation during oxidative stress and 53BP1-foci formation during downstream DNA repair process. Thirdly, loss of ATM -in murine photoreceptors- protract DNA repair but also promote their survival.

**Conclusions:** We propose that due to high oxidative stress and accumulation of RNA:DNA-hybrids in photoreceptors, expression of ATM is tightly regulated to prevent PCD. Inefficient regulation of ATM expression could be central to PCD and inhibition of ATM-activation could suppress PCD in retinal dystrophy patients.

## Introduction

**Figure 1.**
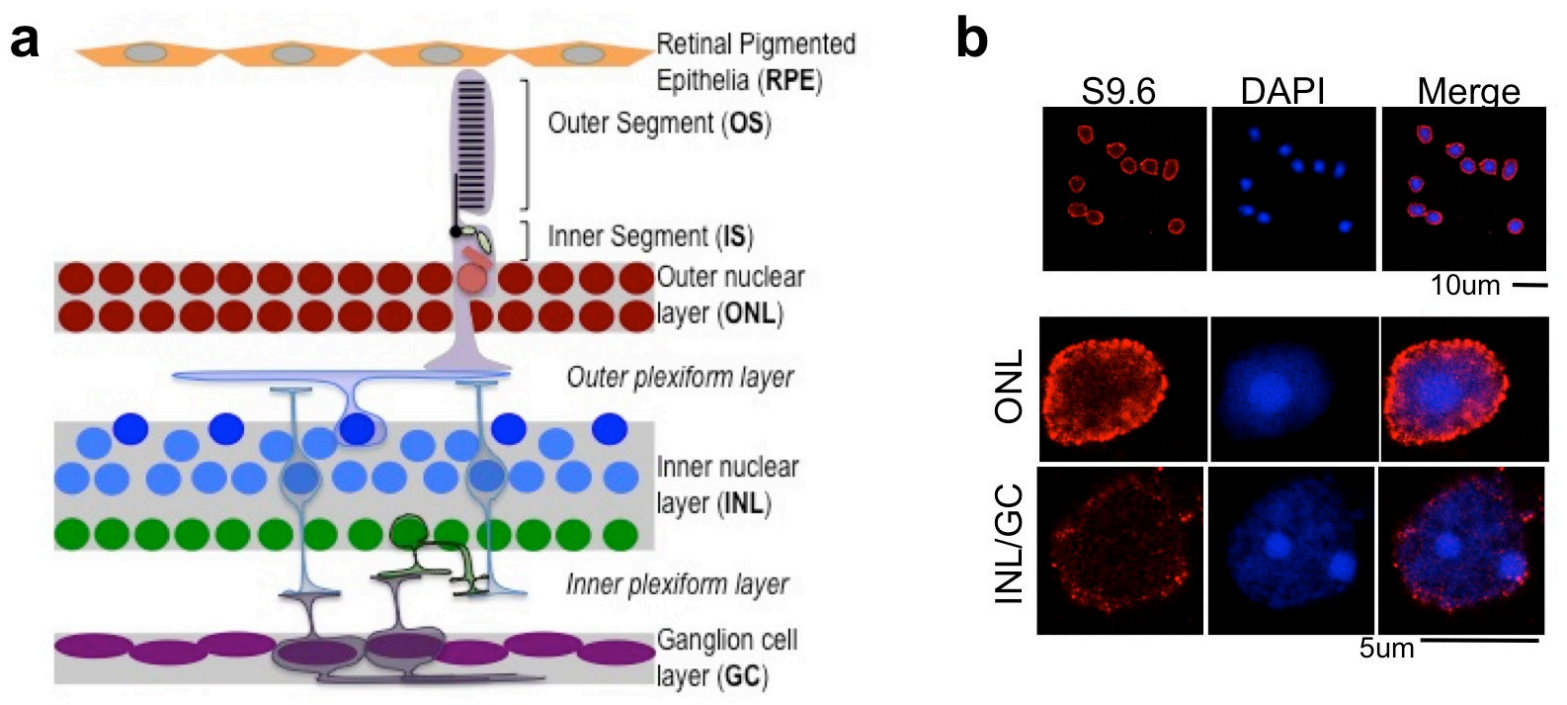
RNA:DNA-hybrids accumulate in photoreceptor nuclei. ( **a**) Labelled diagram shows stratified organization of retinal layers and cell types. ( **b**) Immunofluorescence with DNA:RNA hybrid specific S9.6 antibody of mice retinal cells. Tissue was proteolysed and disintegrated for staining (see Methods). Photoreceptors (outer nuclear layer cells) can be identified by typical inverted chromatin, as seen by DAPI stain.

Photoreceptors are light-sensory neurons and one of the six major cell types in the retina, which are organized into stratified layers ( Figure 1a). Mutations in more than 250 genes, both retina-specific and ubiquitously expressed, are associated with inherited retinal dystrophies (IRDs) ( https://sph.uth.edu/retnet). These genes have approximately 20 known cellular functions ^1^. Mutations in genes coding for proteins involved in retina-specific functions (i.e., phototransduction, visual cycle, retinal development, etc.) could well explain photoreceptor dysfunction or degeneration. But, the reasons that mutations in ubiquitously expressed genes can result in PCD remains unresolved.

A prime example is mutations in ubiquitously expressed members of the U4/U6-U5 tri-snRNP particle (PRPF31, PRPF3, PRPF4, PRPF6, PRPF8) and splice-complex proteins (SNRNP200 and PAP1), which are the second-most frequent cause of autosomal dominant forms of retinitis pigmentosa (adRP) after mutations in rhodopsin ^1– 3^. An exception is DHX38, a spliceosome complex associated RNA-helicase, which has an autosomal recessive pattern of inheritance ^4^.

Heterozygous mutations in human *PRPF* genes does not affect any cell type but specifically causes PCD. What makes photoreceptor cells more susceptible to mutations in PRPF is presently unknown. Wheway *et al.* reported, using mouse cells, that PRPF6, 8, and 31 are important for ciliogenesis ^5^. Photoreceptor cells are specialized sensory cilia and defects in ciliogenesis can primarily affect photoreceptor biogeneisis and survival, as seen for mutations in genes such as *CEP290* and *BBS* ^1^. However, unlike those in *CEP290*, mutations in PRPF do not cause PCD in mice. Mice knockout models of *PRPF31*, *PRPF3* and *PRPF8*, as well as knock-in models containing analogous mutations, do not show any photoreceptor degeneration ^6, 7^. It was also hypothesized that PRPF-mutations could have more pronounced effect on the splicing of photoreceptor-specific genes, thus specifically deteriorating the health of photoreceptors. As observed, haploinsufficiency of PRPFs causes genome-wide splicing defects and does not explain the photoreceptor-specific phenotype ^1, 8, 9^. Evidently, some other factor or combination of factors causes the higher vulnerability of photoreceptors to the loss of splicing proteins.

Inefficient splicing or defects in mRNP biogenesis can lead to genomic instability by an RNA:DNA-hybrid-dependent mechanism ^10, 11^. RNA:DNA-hybrids are formed by re-hybridization of nascent RNA with negatively supercoiled DNA behind the moving RNA polymerase, and plausibly accompanied by a single-stranded non-template DNA, to form a three-stranded structure known as R-loop ^11, 12^. RNA:DNA hybrids are shown to cause DNA breaks in replication-dependent as well as replication-independent manner, mainly by impeding transcription and replication progression ^11, 13, 14^.

Although RNA:DNA hybrids have not been observed in post-mitotic neurons; their role in neurodegeneration is alleged ^15^. Many proteins involved in RNA:DNA-hybrid dissolution are associated with neurodegeneration. RNA:DNA-hybrid helicases such as senataxin (SETX) and aquarius (AQR), are associated with ataxia with oculomotor apraxia type 2 (AOA2) and type 1 (AOA1) ^16– 18^. Nucleotide excision repair proteins, ataxia telangiectasia mutated (ATM) and Fanconi anaemia pathway proteins, which are associated with neurodegeneration, neurodevelopmental defects or microcephaly, have recently been implicated in RNA:DNA-hybrid resolution ^13, 14, 19, 20^. This led us to speculate that RNA:DNA hybrids could be formed in retinal neurons and could play a role in retinal degeneration.

## Results

### RNA:DNA hybrids specifically accumulate in photoreceptor cells of retina

We checked if post-mitotic retinal neurons could accumulate RNA:DNA hybrids. Mice retina was stained with S9.6 (RNA:DNA-hybrid-specific) antibody. Higher levels of RNA:DNA-hybrids are observed in adult photoreceptor nuclei than in the other retinal neurons ( Figure 1b). Nuclei of murine photoreceptors have an inverted chromatin organization, with central heterochromatin and peripheral euchromatin ( Supplementary Figure 1a). Interestingly, the RNA:DNA-hybrids are observed on the peripheral euchromatin region of photoreceptor nuclei, in proximity to the nuclear membrane ( Figure 1b, middle panel). We also checked the expression of RNAseH1 (a ribonuclease) and Senataxin (a helicase), which are enzymes involved in dissolution of RNA:DNA hybrids. Senataxin is expressed mainly in the outer nuclear layer (ONL) of the retina and localized to the euchromatin area of photoreceptor nuclei ( Supplementary Figure 1b). Contrastingly, RNaseH1 is mainly expressed in the ganglion cell layer (GC) and inner nuclear layer (INL) of the retina, but not in the ONL ( Figure 1a and Supplementary Figure 1c). Extra-nuclear staining of RNAseH1 (likely mitochondrial) in the photoreceptor inner segment is observed.

### Loss of PRPF31 cause genomic instability but not in retinal neurons

As photoreceptors can accumulate RNA:DNA hybrids, we wondered whether loss of spliceosomal proteins could lead to RNA:DNA-hybrid-dependent genomic instability in photoreceptor cells. Of the eight PRPFs *, PRPF31* gene aberrations are a major cause of adRP (i.e. RP11) and lead to genome-wide splicing defects ^8, 9^.

**Figure 2.**
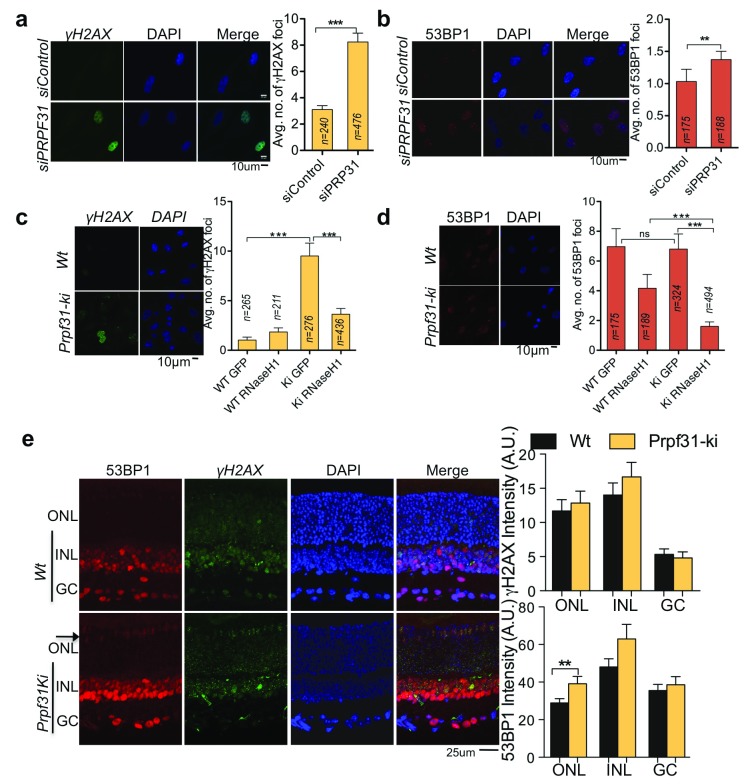
Loss of functional PRPF31 induce RNA:DNA hybrid dependent genomic instability but not in mice retinal neurons. ( **a** and **b**) γH2AX and 53BP1 foci analysis in PRPF31 siRNA-transfected RPE-1 cells. (c and d) γH2AX and 53BP1 foci analysis in vasculo-stromal fraction derived primary cells from *Prpf31 ^+/A216P^* mice ( *Prpf31-ki)*. ( **e**) γH2AX and 53BP1 foci analysis in retina from *Prpf31 ^+/A216P^* mice on postnatal day 20. All column bars represent the mean. For ( **a**- **d**) “n”, mentioned on respective column, signify number of cells analyzed from two independent experiments. For ( **e**) n=16 for each column and signify number of retinal sections analyzed; acquired from n=4 eyes. Error bars represent Standard error of Mean (SEM). *P≤0.05; **P<0.01, ***P<0.001 using Mann-Whitney test ( **a**, **b**), Kruskal-Wallis test followed by Dunn’s post hoc test ( **c**, **d**); and two tailed unpaired Student’s t-test ( **e**).

We used siRNA-based PRPF31 downregulation in RPE-1 cells and quantified foci formation of early DNA damage and repair markers, i.e. γH2AX (H2AX phosphorylated at ser139) and 53BP1. A significant increase in γH2AXand 53BP1 foci is observed in cells depleted of PRPF31 ( Figure 2a, b and Supplementary Figure 2a). We next analyzed primary cells from the stromal vascular fraction (SVF) of PRPF31-deficient mouse models ( *Prpf31* ^+/-^ and *Prpf31 ^+/A216P^*) ^6, 7^. The *Prpf31-A216P* variant reduces the stability and nuclear localization of U4/U6-U5 tri-snRNP complex ^21^. Primary SVF cells obtained from heterozygous *Prpf31 ^+/A216P^* mice show clear accumulation of γH2AX ( Figure 2c, d). Notably, expression of active RNaseH1 in these cells significantly reduced both γH2AX and 53BP1 signal. This indicates the role of RNA:DNA hybrids in genomic instability observed in the absence of functional PRPF31. Cells obtained from Prpf31 ^+/-^ mice also exhibit accumulation of γH2AX ( Supplementary Figure 2b).

We next assessed whether PRPF31-deficient photoreceptors also show increased genomic instability. But unlike RPE-1 and primary SVF cells; no elevation in genomic instability was observed in the retinal neurons of adult *Prpf31 ^+/A216P^* mice (data not shown). In the retina of postnatal day 20 mice, an increase in γH2AX and 53BP1 foci was observed ( Figure 2e). Notably, 53BP1 is not expressed in the ONL (composed of photoreceptor nuclei), except in the apical (outermost) layer of photoreceptors nuclei ( Figure 2e, arrow).

### Photoreceptor cells show slower DNA repair, independent of ATM and 53BP1

**Figure 3.**
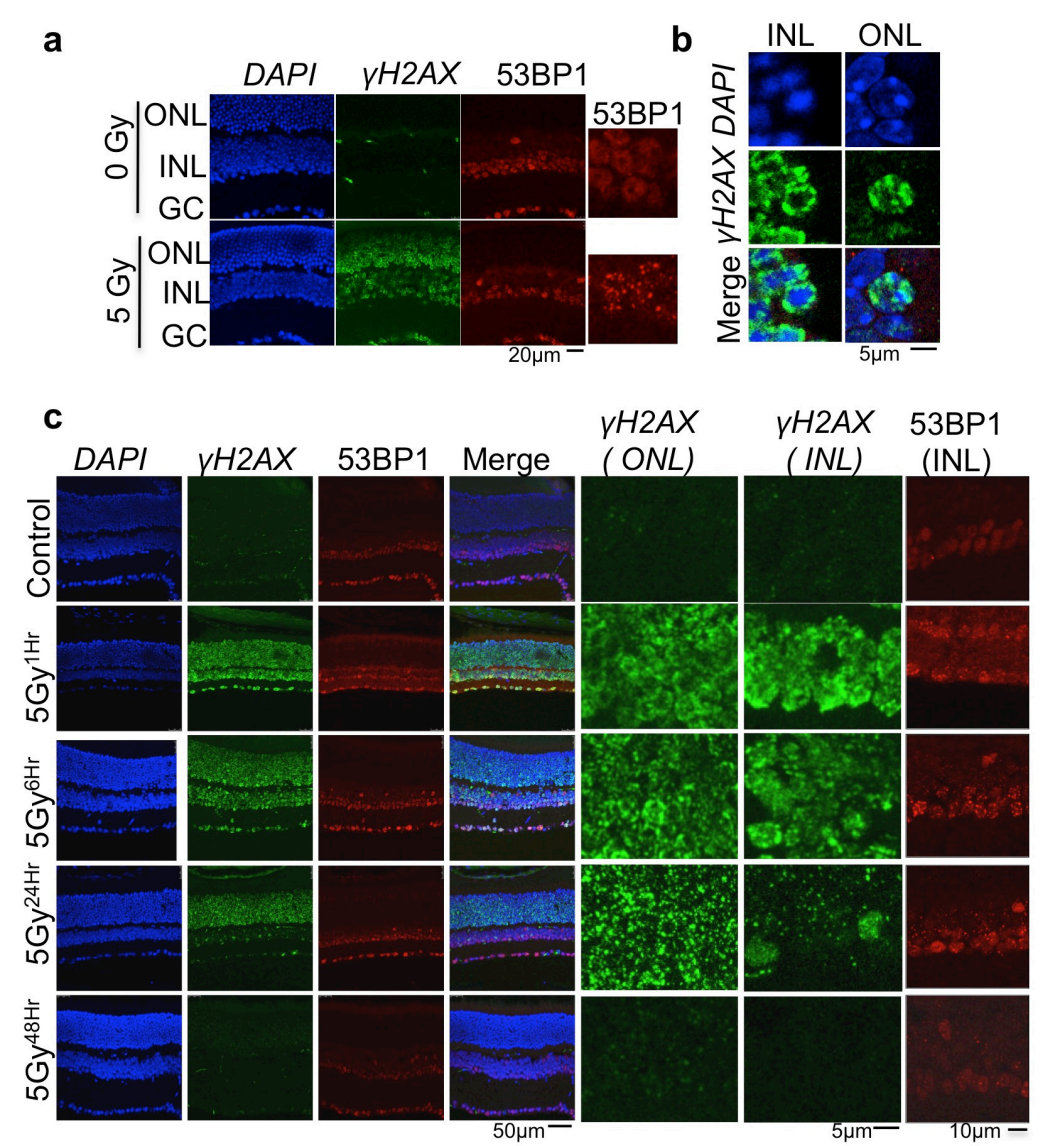
DNA repair response to irradiation-induced DNA-breaks in adult mice retina. ( **a**) Immunofluorescence performed using anti-γH2AX and 53BP1 antibodies in mice retina 1hr after exposure to 5 Gyrase of ionizing radiation. γH2AX appears in all cell types in response to DNA breaks. 53BP only observed in the ganglion cell layer (GC) and inner strata of inner nuclear layer (INL) (see Figure 1a). Zoom in show foci formation in cells expressing 53BP1. ( **b**) H2AX phosphorylation in all retinal cell types is euchromatin specific. ( **c**) Kinetics of DNA repair in mice retina. Post-Irradiation, mice were sacrificed at indicated times and retinal sections were analysed for γH2AX and 53BP1. Left panel show all nuclear layers of retina; *right panel* show zoom in images to emphasize foci formation. Two mice were used for each condition, which were processed and stained together. Random image were taken using confocal microscope on each eye.

The fact that adult mouse photoreceptors can accumulate RNA:DNA hybrids, but do not show any accumulation of genomic instability markers is puzzling. To understand why this is the case, we looked at DNA repair markers in irradiated photoreceptor cells. As reported previously ^22^, we also observed that mouse photoreceptor cells have inefficient DNA repair. Irradiation induces γH2AX formation in all retinal cell types, but localizes only to the euchromatin region ( Figure 3a, b). As aforementioned, 53BP1 is not observed in ONL (containing the nuclei of photoreceptors) and the outer half of the INL (composed mainly of horizontal cell nuclei) ( Figure 1a, Figure 3a). Only at 24 h post irradiation did the γH2AX signal disappear from the nuclei of photoreceptors ( Figure 3c). We also checked for irradiation-induced cell death by terminal deoxynucleotidyl transferase dUTP nick-end labeling (TUNEL) assay. The retinal neurons show resistance to irradiation-induced cell death and, unlike the ganglion cell layer, ONL showed no TUNEL-positive cells until 24 h post-irradiation ( Supplementary Figure 3).

**Figure 4.**
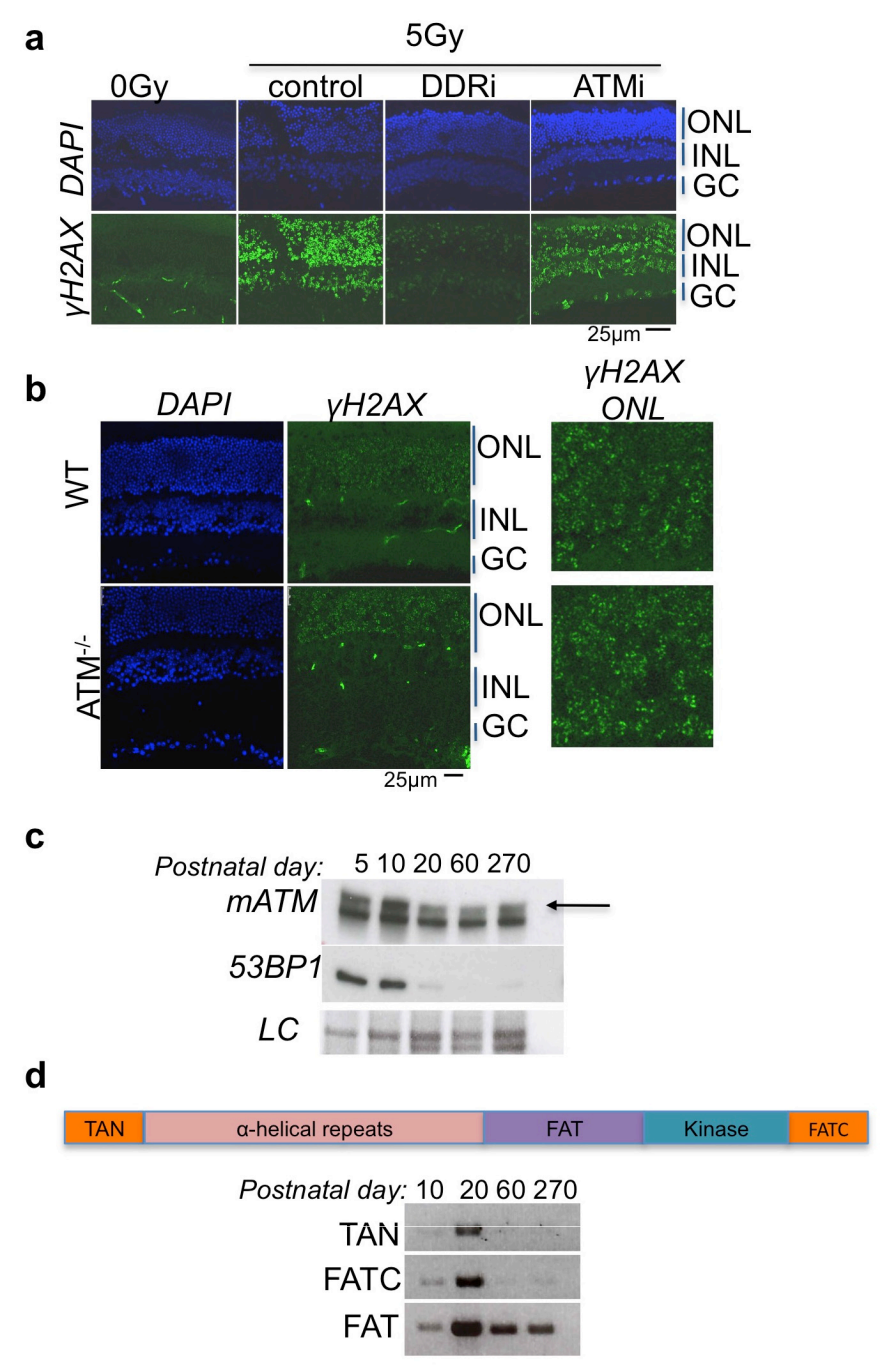
Loss of ATM function in retina. **( **a**)** Retinal explants pretreated with ATM inhibitor (ATMi) or PI3K inhibitor (DDRi) are irradiated and sections analyzed for γH2AX. ( **b**) ATM knockout adult mice retina analyzed for irradiation dependent γH2AXaccumulation. ( **c**) Western blot analysis of ATM and 53BP1 in micro-dissected neuronal retina. LC, Coomassie-equivalent staining of gels used as loading control (representation of n=2 independent repeats). ( **d**) Cartoon depiction of domains of ATM protein. ( **e**) Neural retina of mice was microdissected and cDNA was prepared. PCR performed using indicated domain-specific primers on *ATM* mRNA. Unlike FATC and TAN domain coding mRNA; the FAT-domain containing mRNA could be observed even in 5-month-old adult mice retina, indicating the presence of an alternatively spliced form of ATM in the retina.

ATM is a major PI-3 kinase for post mitotic neurons. We observed that in photoreceptors, most of irradiation-induced H2AX phosphorylation is independent of ATM ^22^. Absence of functional ATM or the presence of ATM inhibitor does not inhibit H2AX phosphorylation in the ONL of the retina ( Figure 4a, b). Consistently, western blot analysis of micro-dissected neural retinas showed that 53BP1 and ATM levels were depleted around post-natal day 20 ( Figure 4c). Although analysis of cDNA from the neural retina shows that an alternative spliced form of *ATM*, lacking the N-terminal PRD and C-terminal FATC domains, could be present ( Figure 4d).

### RNA:DNA hybrids effectuate ATM-activation in presence of oxidative stress

Inefficient DNA repair and an absence of ATM is not expected in photoreceptors, especially considering that markers of oxidative stress are most pronounced in the cerebellum and retina ^1, 23, 24^. Oxidative DNA damage is the most common cause of DNA damage in post-mitotic neurons and could result in single-strand breaks, a major cause of neurodegeneration. Antioxidant treatments have proved to be promising neuroprotective strategies for many retinal dystrophies ^25, 26^.

ATM is a sensor of oxidative stress ^27, 28^. DNA topology or subtle chromatin changes could also activate ATM, even in absence of a DNA break ^29^. Activated ATM can signal DNA repair, cell cycle arrest and also cell death mainly by a p53 dependent pathway ^30, 31^. Noticeably, ATM is shown to promote RNA:DNA hybrid formation on transcribed sites by removal of spliceosomal complex ^32^. We wondered if an absence of ATM is linked to the presence of RNA:DNA hybrids and high oxidative stress in mice photoreceptors.

**Figure 5.**
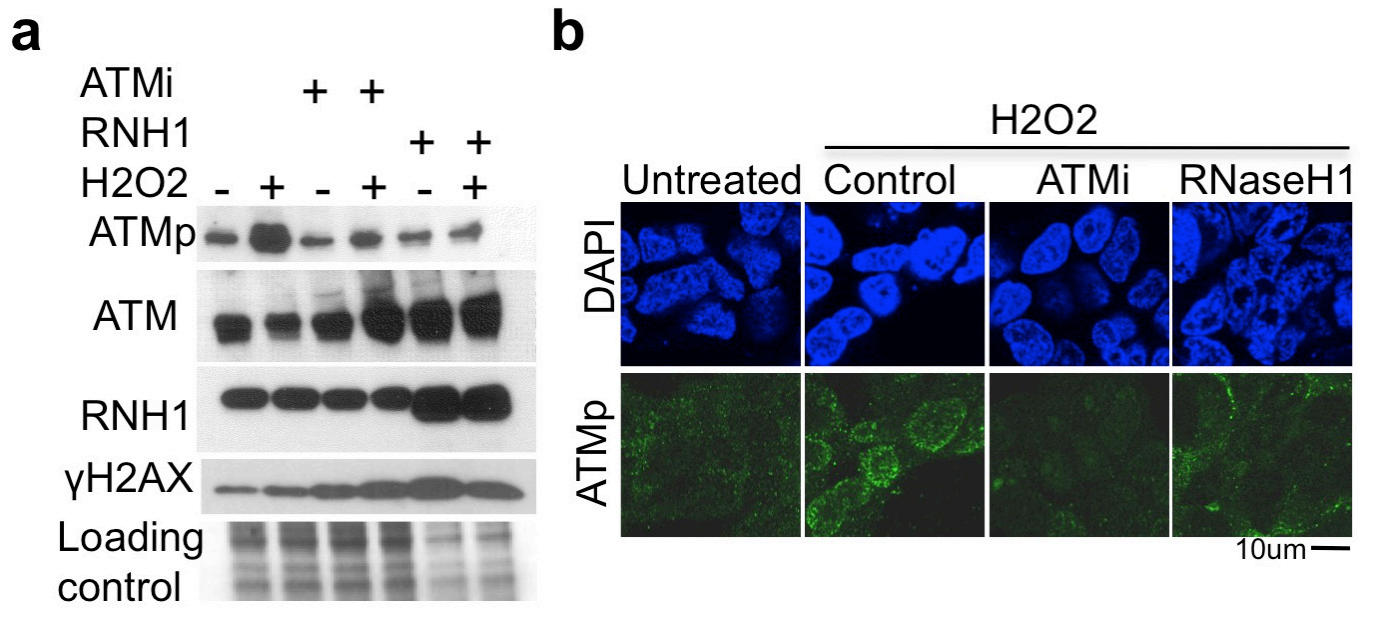
RNA:DNA hybrids promote ATM activation. ( **a**) Western blot of cell extracts treated with H _2_O _2_ in presence of ATM inhibitor or ectopic RNaseH1 expression. Loading control is Coomassie-equivalent staining of gels, before transfer (detailed in the Methods). ( **b**) Immunofluorescence of cells with antibody against ATM phosphorylated on Ser1981. Images are representative of n=4 (for a), and n=3 (for b) independent experiments.

To assess whether RNA:DNA hybrids can directly affect ATM function during oxidative stress; we looked at H _2_O _2_ induced ATM activation in presence and absence of ectopically expressed RNAseH1. Notably, removal of RNA:DNA hybrids by RNaseH1 overexpression completely abolishes ATM phosphorylation at Ser1981 after H _2_O _2_ treatment, as observed by western blotting and immunofluorescence ( Figure 5a, b). The suppression was stronger than that obtained using an ATM-specific inhibitor. We also observed that the H _2_O _2_-induced activation of ATM was more prominent in proximity to nuclear membrane ( Figure 5b).

As ATM activation depends on chromatin association and release, we quantified nuclear ATM in detergent-permeabilized cells expressing inactive hybrid-binding (HB) domain (which stabilizes RNA:DNA hybrids) or active RNaseH1 (which destabilizes the hybrids) ^13^. The results show high variability, with possibly multiple factors controlling the association of ATM with chromatin; however, it seems that stabilization of hybrids could increase the nuclear retention of ATM ( Supplementary Figure 4a). The amount of ATM cross-linked to insoluble pelleted chromatin increased after H _2_O _2_ treatment and could be partially released by over-expressing RNAseH1 ( Supplementary Figure 4b). This suggests that RNA:DNA hybrids regulate the interaction of ATM with chromatin.

### RNA:DNA hybrids co-localize and promote 53BP1 foci formation

**Figure 6.**
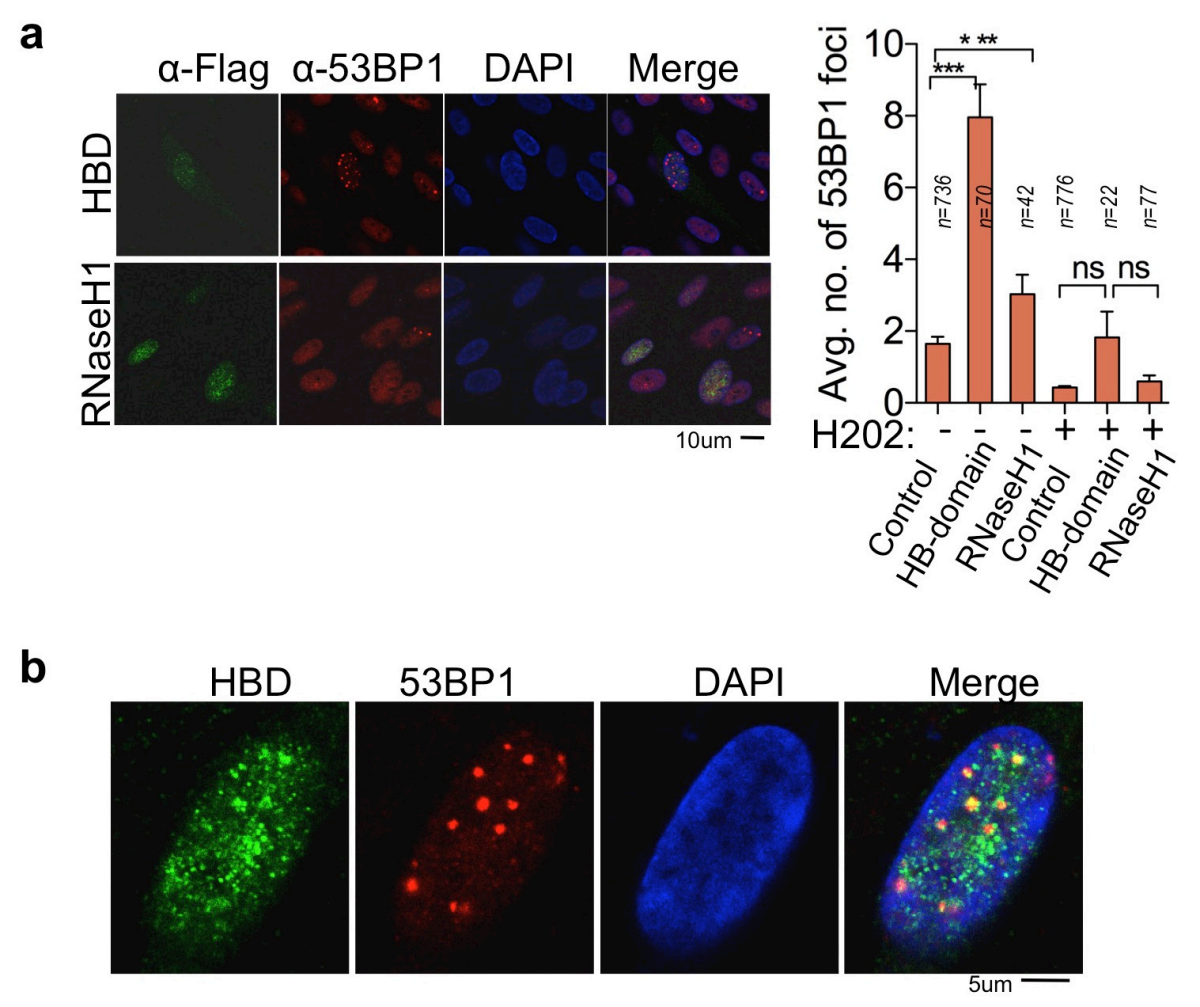
RNA:DNA hybrids promote 53BP1 foci formation. ( **a**) Immunofluorescence using the anti-flag and anti-53BP1 antibody in cells expressing Flag-tagged hybrid-binding (HB) domain or flag-tagged active RNaseH1. 53BP1 foci were quantified in cells expressing HB-domain or RNaseH1. Column bars represent the mean of n number of cells (described on each column), from three independent experiments. Error bars represent SEM. *P ≤ 0.05; **P < 0.01, ***P < 0.001 using the Kruskal-Wallis test followed by Dunn’s post hoc test. ( **b**) Higher zoom representative images show HB-domain and 53BP1 co-localization, specifically in the euchromatin area.

We next looked at 53BP1, which is also absent from photoreceptors. 53BP1 is a pro-non-homologous end-joining protein and accumulates on damaged DNA sites in an ATM-dependent manner ^33, 34^. The formation of 53BP1 foci is crucial for DNA repair, checkpoint activation and cell death ^33, 35, 36^. Notably, stabilization of RNA:DNA hybrids via the expression of the HB domain significantly increases 53BP1 foci formation. Conversely, RNAseH1 overexpression significantly suppresses 53BP1 foci formation ( Figure 6a).

Notably, high-resolution images using confocal microscopy showed that 53BP1 foci consistently co-localize with RNA:DNA hybrids ( Figure 6b), indicating the clear affinity of 53BP1 for chromatin regions containing RNA:DNA hybrids. However, unlike ATM, H _2_O _2_ treatment does not increase the number of 53BP1 foci, but stabilization of RNA:DNA hybrids by the HB domain increases 53BP1 foci formation, even in the presence of H _2_O _2_ ( Figure 6a, bar graph).

### RNA:DNA hybrids promote ATM-mediated DNA repair

**Figure 7.**
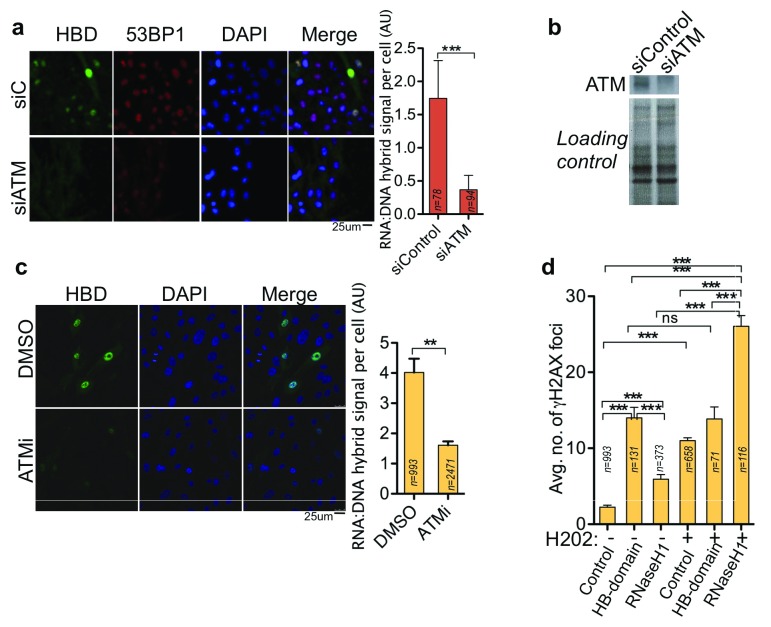
RNA:DNA hybrid formation is central to ATM-53BP1 repair pathway. ( **a**) Immunofluorescence using the anti-flag to quantify nuclear hybrid-binding (HB) domain foci signal in pre-permeabilized RPE cells and quantification of HB domain signal in cells treated with siATM, ( **b**) Western blot showing ATM depletion. ( **c**) Immunofluorescence and HB-domain foci quantification in cells treated with ATM inhibitor. ( **d**) Quantitation of γH2AX foci in cells expressing HB domain or active RNAseH1, in presence or absence of H _2_O _2_ dependent oxidative stress. siC, non-targeted control siLuciferease RNA. Column bars represent the mean of n number of cells (described on each column) from three independent experiments. Error bars represent SEM. *P ≤ 0.05; **P < 0.01, ***P < 0.001 using Mann-Whitney test ( **a**, **c**) and Kruskal-Wallis test followed by Dunn’s post hoc test ( **d**).

Clearly, DNA repair activity of ATM and 53BP1 depend on RNA:DNA hybrids. We next assessed whether ATM or 53BP1 are crucial for RNA:DNA-hybrid removal. Using the HB domain, we probed and quantified RNA:DNA-hybrids in cells depleted of ATM. Notably, the number of RNA:DNA hybrids decrease in cells depleted of ATM ( Figure 7a, b). As shown before, ATM promotes RNA:DNA hybrid accumulation ^32^. This action require ATM phosphorylation, as ATM inhibitor treatment suppresses RNA:DNA hybrid formation ( Figure 7c). Expectedly, ATM-depleted cells are also inefficient in forming 53BP1 foci ( Figure 7a). The results supports the idea that the ATM-dependent 53BP1 association with chromatin and RNA:DNA hybrid formation are interdependent events.

RNA:DNA hybrids are primarily considered to be a source of genomic instability. Notably, we observe higher levels of oxidative stress-dependent genomic instability when RNA:DNA hybrids are removed. In Figure 7d, as expected, stabilization of RNA:DNA hybrids by the HB domain could increase γH2AX foci levels. However, when treated with H _2_O _2_, HB-domain expressing cells do not show further increases in γH2AX foci. Contrastingly, destabilization of RNA:DNA hybrids by RNaseH1 overexpression results in manifold γH2AX foci accumulation after H _2_O _2_ treatment. Similar results are obtained in western blot analysis, wherein the cells over-expressing RNAseH1 show increase in γH2AX after H _2_O _2_ treatment ( Figure 6a). Very likely, RNA:DNA hybrid formation is a crucial step during ATM-mediated DNA repair, and higher levels of γH2AX in the absence of ATM-activation is a result of prolonged DNA repair and damage accumulation.

### Absence of ATM slow down photoreceptor cell death in rd1 mice

ATM is crucial for repair as well as induction of apoptosis. In the presence of unrepaired DNA breaks or blocked DNA-protein complex intermediates, ATM can initiate signaling of the cell death pathway ^31^. ATM-deficient cells are defective in irradiation-induced apoptosis ^30^. As aforementioned, the ONL of the mouse retina shows resistance to irradiation-induced cell death, as observed by TUNEL staining ( Supplementary Figure 3). ATM-induced cell death is via p53 dependent pathway ^31^. It is known that ectopic expression of p53 in photoreceptor cells promotes photoreceptor cell death ^37^, although the mechanism is unclear ^38^.

We observed that stabilization of hybrids by the HB domain in RPE-1 cells leads to S-phase accumulation possibly by RNA:DNA-hybrid-dependent inhibition of replication fork progression ( Supplementary Figure 5) ^10, 11^. Notably, unlike RPE-1 cells, HB-domain expression in HT1180 cells shows an accumulation of sub-G1 apoptotic cells, but no defects in S or G2 phase progression ( Supplementary Figure 5). The different outcomes show that stable RNA:DNA hybrids not only create replication stress but can also induce cell death, possibly via the apoptotic pathway.

Photoreceptors are post-mitotic terminally differentiated neurons, thus RNA:DNA-hybrid-induced replication stress cannot occur. However, in the presence of high oxidative stress in photoreceptors, RNA:DNA hybrid dependent constitutive ATM activation could promote cell death ^30, 31^. We thus wondered whether the absence of ATM in retinal neurons promotes photoreceptor cell survival during high oxidative stress and metabolic demands.

**Figure 8.**
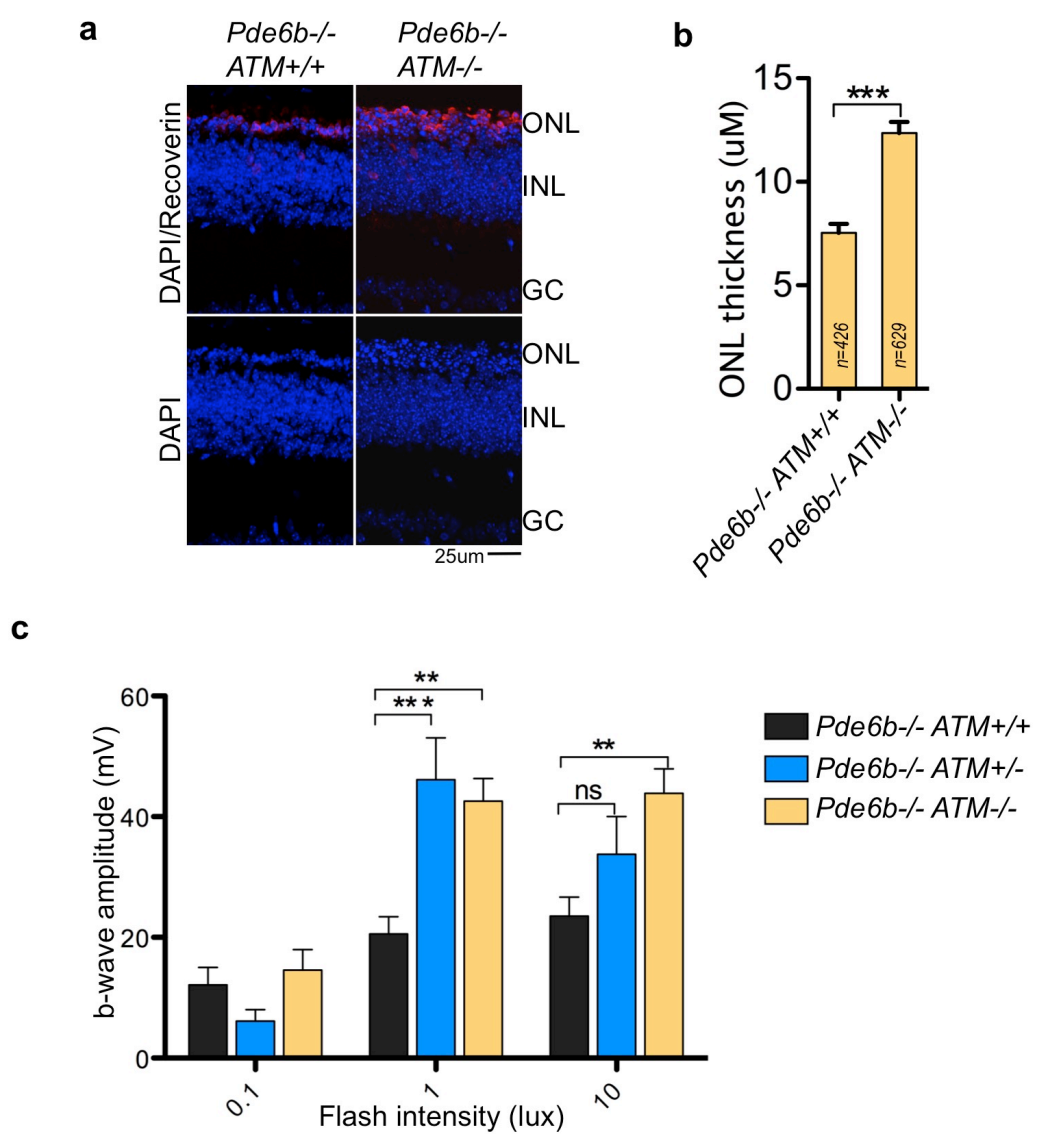
Loss of ATM promotes photoreceptor survival. ATM expression was knockdown in rd1 background and postnatal day 20 mice retina were analyzed by different methods. ( **a** and **b**) Immunofluorescence using the anti-Recoverin antibody performed on retinal sections of Pde6b ^-/-^ ATM ^+/+^ and Pde6b ^-/-^ ATM ^-/-^ mice. Line scan analysis was performed and outer nuclear layer thickness was quantified using Recoverin staining (detailed in Methods). Column bars represent Mean for, “n” number of line scans, performed on n=32 sections from n=2 Pde6b ^-/-^ ATM ^+/+^ and n=3 Pde6b ^-/-^ ATM ^-/-^ animals. ( **c**) Electroretinogram of mice retina was performed in dark adapted animals and b-wave was quantified. Column bars represent mean for n=18 electroretinogram (ERG) readings from n=9 animals for each Pde6b ^-/-^ ATM ^+/+^ and Pde6b ^-/-^ ATM ^-/-^ genotype; and n=12 ERG readings for n=6 mice with Pde6b ^-/-^ ATM ^-/+ ^genotype. Error bars represent SEM. *P ≤ 0.05; **P < 0.01, ***P < 0.001 using Mann-Whitney test ( **b**) and one-way-ANOVA followed by Turkey test ( **c**).

As ATM is only expressed before postnatal day 20 in the mouse retina ( Figure 4c, d), we used the rd1 mouse model, which has a mutation in Phosphodiesterase 6B gene and loses 80 percent of photoreceptors before postnatal day 15 due to severe oxidative stress ^39^. We removed ATM in the *Pde6b ^-/-^* background and analyzed the retina of the animal at p20. In the retina of ATM knockout mice ( *Pde6b ^-/-^ Atm ^-/-^)*, photoreceptor cell death decelerates and the thickness of the ONL is significantly higher ( Figure 8a, b).

We next resorted to electroretinogram (ERG)-based functional analysis of the retina, which measures the light-induced trans-retinal flux of ions. ERG of *Pde6b ^-/-^ Atm ^-/-^* mice shows a slight but significantly improved b-wave in dark-adapted animals, indicating the protection of retinal function, as compared to *Pde6b ^-/-^ Atm ^+/+^* animals ( Figure 8c).

We next assessed what happens in human photoreceptors. As observed by S9.6 antibody staining, RNA:DNA hybrids are also observed in human photoreceptor nuclei, though unlike mice photoreceptors they are in central euchromatin region ( Supplementary Figure 6a). Notably, unlike the murine photoreceptors, expression of ATM and 53BP1 in adult human retina is observed by immunofluorescence ( Supplementary Figure 6b). All data are available on OSF ^40^.

## Discussion

RNA:DNA-hybrid-dependent ATM hyper-activation during oxidative stress could promote photoreceptor cell death. The mechanism could be more pronounced in human patients but not murine RP models, as former express higher levels of ATM and 53BP1. This could possibly explain why some mouse models, e.g. *Prpf31* mutants *,* which cause retinitis pigmentosa-11 in humans, do not exhibit photoreceptor cell death ^1, 7^. Detailed studies are required, but we anticipate that expression of ATM and 53BP1 are fine-tuned in photoreceptors with respect to RNA:DNA-hybrid and oxidative stress levels ( Supplementary Figure 7). Loss of this equilibrium by increased RNA:DNA-hybrid accumulation or higher expression of ATM could cause PCD in progressive retinitis pigmentosa and age-related macular degeneration ^1, 4^. Loss of ATM and 53BP1 in mice photoreceptors is a compromise in which decreased sensing for DNA damage is coupled to slower activation of cell death signaling in post-mitotic neurons ( Supplementary Figure 7). Possibly, unlike mice, humans are diurnal and live much longer, thus would require better DNA repair, and have tighter uncoupling of gene-expression and DNA repair. It was recently reported that osteoclast cells also show better survival in absence of ATM ^41^. Though there must be additional factors that influence photoreceptor cell survival, we think our work will help to improve understanding of the mechanism underlying photoreceptor cell death, and propose ATM and 53BP1 as targets of neuroprotection strategies in the human retina.

The molecular mechanisms that sense and resolve RNA:DNA hybrids are unclear and under intense investigation ^5, 6^. It is known that RNA:DNA hybrids are intimately linked to genomic instability and replication stress. Our work shows that formation of RNA:DNA hybrids is central to the ATM-53BP1-dependent repair pathway. Thus, RNA:DNA hybrids could affect the efficiency of DNA repair, cell death signaling and checkpoint activation ( Supplementary Figure 7). We expect that future work to understand RNA:DNA-hybrid-associated molecular pathways will further elucidate its role in neurodegeneration and ageing.

## Methods

### Cells and cell culture

Primary vascular stromal fractions (VGF) were obtained by standard protocol. Confluent cultures of primary VGF cells, RPE-1, HCT1180 and HEK293T cells were all maintained in DMEM (#32430027, Gibco™) supplemented with 10% FBS, at 37°C and 5% CO _2_. To induce oxidative stress, cells were incubated in DMEM containing freshly diluted 500 µM H _2_O _2,_ for 1 h at 37°C and 5% CO _2_, before being processed for western blot or immunofluorescence.

### siRNA-based depletion

Sequences of siRNA used were: siLuciferase (negative control), 5′-CGUACGCGGAAUACUUCGA-3′; siATM, 5′-GACUUUGGCUGUCAACUUUCG-3′; and siPRP31, 5’-AGGAUGAGAUCGAGCGCAA-3′. Cells were transfected using Oligofectamine™ Transfection Reagent (#12252011, Invitrogen™) by manufacturer-defined protocol in Opti-MEM™ (#31985070, Gibco) cell culture media and incubated for 48 h before being processed for immunofluorescence. 

### 3Xflag tagged HB-domain AAV viral DNA constructs and RNA:DNA hybrid probing

3X flag tagged HB domain of RNaseH1 and 3Xflag tagged full RNaseH1 lacking the mitochondrial targeting sequence ^13^ were cloned into a pAAV-IRES-hrGFP (Agilent; #240075) plasmid and viral particles were produced using the AAV Helper-Free System (Agilent #240071), as per the manufacturer’s instructions. Cells were transduced in Biosafety level 2 facility and incubated for at least 24 h to allow expression of the tagged protein. For siRNA-based experiments, GFP-positive transduced cells- expressing RNaseH1 or HB domain were FACS-sorted using a BD FACS Aria ^TM^ cell sorter (#P-07900125; BD Biosciences), equipped with FACSDiva software (V5.0.3; BD Biosciences). Cells were cultured and transfected as aforementioned. siRNA-treated cells were analyzed 48h after transfection. To study the effect of ATM inhibition in RNA:DNA hybrid accumulation, cells were incubated with complete DMEM containing 10 µM ATM inhibitor (KU55933; Tocris), for 24 h. The plates were light protected and inhibitor-containing medium was replaced every 8 h.

### Immunofluorescence

The primary antibodies and dilutions used were: anti-gH2AX (Clone JBW301, 05-636-Merk Millipore; source, mouse; 1:100), anti-53BP1 (NB100-304, Novus biologicals; source:Rabbit, 1:100) ATM (Sigma, MAT3-4G10/8; source, mouse; 1:200) ATM pSer1981 (Cell Signaling, #4526; source, mouse; 1:100), anti-RNaseH1 (Proteintech, #15606-1-AP, source, rabbit; 1:100) anti-Recoverin (Millipore, AB5585; source, rabbit; 1:500), anti-Flag (Sigma, M2 clone; source, mouse, 1:1,000), anti-RNA:DNA hybrid (S9.6 clone, a kind gift from Andres Aguilera lab and later bought from Kerafast # ENH001; source, mouse; 1:50). (Note that the S9.6 antibody show variation in staining efficiency and care should be taken by adding a only-secondry-antibody control). When used in combination with anti-Flag antibody (to detect Flag-tagged proteins), anti-gH2AX (Cell Signaling, #2577; source, rabbit; 1:100) and anti-ATM (Santa Cruz, #sc7129-Q19; source, goat; 1:50) was used. AlexaFluor® (Molecular Probes) secondary antibodies were used, conjugated to green (488), red (555) and far-red (633) fluorophores (donkey anti-mouse, #A21202; donkey anti-rabbit, #A21206; goat anti-Mouse, #A21422; goat anti-rabbit, #A21070; goat anti-mouse, #A21052), all at a dilution of 1:400. Acquisition settings were adjusted using primary and secondary antibody controls, to rule-out any cross-channel signal detection and autofluorescence. Cells grown on glass coverslips were fixed for 10 min in 2% methanol-free formaldehyde (Sigma) in PBS. Cells were washed and permeabilized with 70% ethanol (20 min at -20°C) and stored in 70% ethanol at 4°C. To stain, cells were blocked for 30 min in blocking solution (PBS with 0.1% Triton X-100 and 5% BSA), followed by overnight primary antibody in blocking solution, washed and 1 h in secondary antibody in blocking solution. After three washes in excess PBS 0.1% Triton X-100, coverslips were blot-dried and mounted in 4,6-diamidino-2-phenylindole (DAPI) containing Vectashield mounting media (Vector Labs H-1201). Images were acquired on Leica TCS SP5 Confocal microscope, equipped with LAS AF software version 2.1 and four lasers sources: 405-Diode, 543 HeNe, 633 HeNe and Argon.

### Immunofluorescence of the mouse retinal sections

For immunofluorescence of the mouse retina, animals were euthanized by cervical dislocation and eyes excised and fixed in 4% paraformaldehyde in PBS for 30 min at room temperature. After repeated PBS washes, fixed eyes were incubated at 4°C for 8 h each in 10% sucrose-PBS, 20% sucrose–PBS and 30% sucrose–PBS followed by 50-50 solution of 30% sucrose and optimal cutting temperature (OCT) compound (Tissue-Tex,#4583). The eyes were frozen in 100% OCT in dry ice and stored at -20°C. For cryotome sectioning, performed at -20°C, serial sections 18-mm thick were mounted in five parallel series and stained as described for cells above. For RNA:DNA-hybrid detection using S9.6 antibody, sections were pretreated with Accutase (Sigma; #A6964) for 30 min at room temperature for tissue dissociation and staining was performed as reported by Bhatia *et al.* ^13^. For TUNEL assay the *In Situ* Cell Death Detection Kit (Roche, Mannheim, Germany), was used as per manufacturer’s instructions.

### Quantitative analysis of immunofluorescence images

Metamorph (Molecular Devices, Version 7.1) image analysis software was used to quantitate foci, signal intensity and signal area with inbuilt functions, i.e. Granularity and Line Scan. In brief, for foci analysis, maximum projection of z-stacks is created (with LAS AF Leica software) and saved as .tiff images. The images are opened on Metamorph and converted to 8-bit format by inbuilt "Multiply-function". Background subtraction is done based on unstained area in the image. A mask is created, based on DAPI image, to assign the nuclear area. Using the Granularity function, the signal (with minimum granule size in pixels) is quantified per nuclei and updated in a linked excel sheet. To measure ONL thickness in mice retina using metamorph, recoverin-stained retinal section images were opened and three lines per image were drawn perpendicular to the ONL (i.e. recoverin-stained ONL) using the LineScan-tool. The intensity measurements are automatically documented in a excel file. Length of line with pixels containing positive value (for recoverin signal) was used to measure the thickness of ONL. The length in pixels was converted in uM by multiplying with the conversion factor, obtained by measurement of the scale-bar on the image. These measurements were later used for quantitative analysis in GraphPad Prism 5 package software.

### Irradiation induced DNA damage in the retina

Mice were anesthetized by sub-cutaneous injection of ketamine/xylazine (80/12; mg/kg body weight) and exposed to 5 Gy of gamma irradiation (BioBeam-8000: Gamma Service Medical GmbH). After irradiation, mice were returned to their cages for 1 h (unless specified; as in Figure 3c). Thereafter mice were euthanized and the eyes were processed as mentioned before. To pre-treat the retina with small molecule kinase inhibitors, the cornea is dissected out as described by Donovan *et al.* ^42^. Explants were incubated for 1 h in DMEM+10% FBS medium with 10 µM ATMi (KU55933; ATM inhibitor from Tocris) or 5 µM of DDRi (PI3 kinase inhibitor; a gift from Prof. Oscar Fernandez Capatillo, CNIO, Madrid). Explants were then exposed to 5 Gy of gamma irradiation and incubated in DMEM (with ATMi or DDRi) for 1 h at 37°C and 5% CO _2_. Thereafter, the retinal explant were processed as aforementioned for mouse eyes.

### Western blots

Cells were recovered by Accutase (Sigma, #A6964) treatment for 5 min at 37°C. For the neural retina, the two animals for each age group were euthanized by cervical dislocation (postnatal day 10, 20, 60, 270) or by decapitation (postnatal day 5). Eyes were removed and neuronal retina was separated by microdissection as described by Donovan *et al.* ^42^. Cells/tissues were lysed using RIPA lysis buffer (Sigma, #R0278) for 30 min on ice and centrifuged at 4°C (Eppendorf, #5415R) to remove the debris. Protein concentration in supernatant was measured on a Nanodrop ND-100 Spectrophotometer (NanoDrop Technologies). Normalized volume of samples were appropriately diluted with 4X-SDS-PAGE sample buffer, to obtain equal concentration and 1X resultant sample buffer concentration. Samples were heated for 10 mins at 90°C and loaded on Mini-PROTEAN® TGX Stain-Free™ (4–20% gradient) Precast Gels (BIO-RAD, # 456-8096). After electrophoresis, gels were scanned under ultraviolet light to get Coomassie-equivalent staining, which was used as the loading control. Overnight transfer was performed in Tris-Glycine buffer with 5% methanol, onto an Amersham Hybond ^TM^-P 0.45 blotting membrane. After the transfer, the gel was again UV exposed to check the efficiency of transfer. For blocking, PVDF membrane was incubated in SuperBlock™ (PBS) Blocking Buffer (Thermo Scientific™,# 37515) with 0.1% Tween-20, for 30 minutes at room temperature. Primary antibody used were anti-ATM (Sigma, MAT3-4G10/8, 1:1000), anti-ATM pSer1981 (Cell Signaling, #4526, 1:1000), anti-H2AX (Clone JBW301, 05-636-Merk Millipore, 1:500), anti-53BP1 (NB100-304, Novus biologicals, 1:1000), anti-RNaseH1 (Proteintech, #15606-1-AP, 1:1000), anti-β-actin (Sigma,#A3854, 1:1000). Antibodies were diluted in PBS with 0.1% Tween-20 and 2% BSA (Calbiochem,#12659), overnight at 4°C. Anti-mouse and anti-rabbit, HRP-conjugated secondary antibodies (Sigma, #A4416 and #A0545, respectively) were used at 1:20,000 dilution for 1 h at room temperature. Probed PVDF membranes were treated for 5minutes with Western Bright ^TM^ ECL reagent (Advansta, #K12045-D20) and imaged using Amersham Hyperfilm ^TM^ (GE-Healthcare,#28906844) and Hyperprocesor (Amersham Biosciences, Model SRX-101A). To re-probe with another antibody, membranes were stripped using Restore ^TM^ western blot stripping buffer (Thermo Scientific, #21059) for 10 min at room temperature.

### Primers and PCR for ATM expression in neural retina

The neural retina of two mice for each time point (at postnatal days 10, 20, 60 and 270) was microdissected; 1 eye each was used for western blot and one eye was used to prepare total RNA. RNA was quantified using Nanodrop ND-100 Spectrophotometer (Manufactured by NanoDrop Technologies), normalized and reverse transcribed to cDNA using QuantiTect Reverse Transcription Kit (Qiagen,#205311). Primers were designed using the Primer3Plus online tool, on the selected exons of ATM cDNA sequence (from the ENSEMBL database). Primers target a region on ATM mRNA, relative to domains on protein sequence. I.e. betwen exon 2–4 for TAN domain; between exon 62–64 for FATC domain and between exon 13–17 for control FAT domain. The primers (sequence given below) were validated using UCSC *in silico* PCR, and selected if they produce single PCR product from mouse transcriptome sequence and no product from mouse genomic sequence. PCR reaction was performed using MyTaq ^TM^ red DNA polymerase, using standard PCR condition with 54°C of annealing temperature (all primers have have Tm between 58°C and 60°C); for 30 sec and extension at 72°C for 1min, for 30 cycles, for all primers.

Primer sequences are as follows. ATM_FATC_domain: 5’-TGCTGACCATTGTAGAGGTTCT-3’ (forward) and 5’-CAGTTCAGTGTGTATGCGGC-3’ (reverse); ATM_TAN_domain: 5’-AGTGGATAAATTTAAGCGCCTGA-3’ (forward) and 5’-AGCCACTGTTGCTGAGATACT-3’ (reverse); ATM_FAT_domain: 5’-TCTGAAACCCTTGTCCGGTG-3’ (forward) and 5’-AGGACTCATGGCACCAACAG-3’.

### Animal handling

All experiments are performed in compliance with Spanish and European Union laws on animal care in experimentation and approved by the Committee of Animal Experimentation, CABIMER, Seville, Spain. Mice are maintained in Specific Pathogen Free (SPF) conditions and health status is monitored through a comprehensive surveillance program. Cages (4–6 adults per cage), bedding (sawdust) and water (sterilized by autoclaving) and food (irradiated Rodent VRF1) were changed weekly (every Tuesday). Room temperature (21°C) and 12–12 light cycle (6 pm - 6 am) were maintained. Equipment and material that need to enter the SPF zone were decontaminated by hydrogen peroxide vapor. The number of mice used in the study was kept to a minimum and sample size calculations were not performed prior to experiments due to lack of equivalent datasets and information about expected results. Power analysis was performed retrospectively to confirm that the power of statistical analysis is >0.8, at alpha=0.05.

Retinal sections for DNA response analysis are from C57BL/6J, adult wild-type mice (8–10 weeks) both male and female (22±3 g). S.S. Bhattacharya lab at University College London, previously reported PRPF31 mouse models 6, in collaboration with Charles River (France). Mice were procured and transferred to the SPF animal facility of CABIMER, Seville, Spain. Prpf31 A216P/+ mice were on a mixed background of 129S2/Sv (source of stem cell used for mutation incorporation) and C57BL/6J (background used from crossing the chimeric mice). Prpf31 ± knockout mouse has BALB/c, 129S2/Sv and C58BL/6J mixed background; as they were generated by crossing the Prpf31 A216P/ ^+^ mouse with a BALB/c mouse expressing Cre recombinase (BALB/c-Tg(CMV-Cre)1Cgn/J) ^6^. Retinal sections were obtained from 20-day old Prpf31 A216P/+ mice. VSF cells were isolated from adult PRPF31 mouse models (8–12 week). ERG and retinal thickness analysis were done on 20-day old Pde6b ^-/-^ ATM ^+/-^ knockout mice. Pde6b ^-/-^ ATM ^+/-^ were produced by crossing ATM ^+/-^ knockout mouse (originally created in a mixed background (129/SvEv and NIH Black Swiss) ^43^ with Pde6b ^-/-^ mice (FVB/Ncrl, from Charles River, an early onset retinal degeneration strain homozygous for allele Pde6brd1). ATM mice were obtained from Felipe Cortes lab (CABIMER, Seville, Spain) and genotyped as reported before ^43^

To study DNA repair response in irradiated mice, for two independent repeats are performed. For each repeat all animals were used from a single litter of C57BL/6J adult wild-type mice of same-sex (all male or all female).

Weaning was performed for all strains at postnatal day 20. For PRP31 mouse models and Pde6b ^-/-^ATM ^+/-^ mice, which were studied at postnatal day 20 (15±2 g), pups were always with mother. For ERG analysis, 24 h dark adaptation was performed with mother. For Prpf31 A216P/ ^+^ mice retinal sections, two mice each i.e. wildtype and heterozygous A216P/ ^+^ (i.e. four total mice i.e. 8 eyes) are analyzed. For VS fraction cells, two mice for each mice model respective wild-type were used i.e. 8 eyes. Independent cultures were maintained, for up to 10 passages.

For ERG analysis, total 24 mice i.e. 48 eyes (from nine ATM ^+/+^, six ATM ^+/-^ and nine ATM ^-/-^ mice) were used, all coming from three litters. Males and females were not distinguished. Animals were genotyped after ERG analysis was performed to reduce any bias. For morphological preservation, retinal thickness analysis was performed for two Pde6b ^-/-^ ATM ^+/+^ and three Pde6b ^-/-^ ATM ^-/-^ were used (total five mice). Metamorph software-based automated Linescan analysis of recoverin staining was performed for retinal thickness analysis, described above.

The primary result of our study is that molecular function of ATM and 53BP1 depend on RNA:DNA hybrids; and removal of RNA:DNA hybrids completely inhibit ATM activation during oxidative stress. The primary outcome from mice models is the partial preservation of retinal structure and function after ATM-removal in rd1 mice. The additional outcomes are: suppressed DNA repair response and loss of ATM and 53BP1 expression in retinal neurons, comparison with human photoreceptors and presence of RNA:DNA hybrids close to the proximity of nuclear membrane of murine photoreceptor cells. All efforts were made to ameliorate the suffering of animals.

### Electroretinography

Electroretinography was performed using a Color Dome Ganzfeld (Diagnosys LCC, MA, USA) as detailed before by Lourdes *et al.* ^44^. Briefly, to evaluate scotopic vision, mice were dark-adapted overnight and anaesthetized by sub-cutaneous injection of ketamine/xylazine (80/12; mg/kg body weight). A drop each of 10% phenylephrine and 1% tropicamide were used to dilate the pupils of the animal. To detect retinal response, the mouse was placed inside the ColorDome Ganzfeld (Diagnosys LCC, MA, USA) and electrodes were touched on the surface of the corneas, pre-treated with a hydrating agent (1% methylcellulose). A single pulse of white-flash (6500 K) was used for stimulation, with the stimulus strength of 0.1, 1 and 10 lux. An average of 15 responses was made, with an inter-stimulus interval of 15 s.

### Statistical analysis

The indicated statistical tests were performed using GraphPad Prism 5 package. In brief, the Kolmogorov-Smirnov normality test was used to check distribution of data (alpha=0.05). A parametric two-tailed Student’s t-test was used for data with normal distribution; otherwise, a non-parametric Mann-Whitney test was applied. For multiple comparisons, one-way-ANOVA followed by Turkey test was used if data had normal distribution; otherwise a Kruskal-Wallis test followed by Dunn’s post hoc test was used. Statistical significance is marked by one, two or three asterisks, indicating P < 0.05, P < 0.01 or P < 0.001, respectively.

## Data availability

All data associated with this study, including all raw microscopy images and uncropped western blots, are available on OSF: http://doi.org/10.17605/OSF.IO/X3CM7 ^40^. Data are available under the terms of the Creative Commons Attribution 4.0 International license (CC-BY 4.0).

## Supplementary material

**Supplementary Figure 1. Expression of DNA:RNA-hybrid-specific enzymes and chromatin organization in the inner nuclear layer (INL) and outer nuclear layer (ONL) neurons of retina.** Immunofluorescence in adult mice retinal sections. (a) Localization of euchromatin histone marker (H3K4me3) in mouse retinal neurons and highlight inverted chromatin organization in photoreceptor nuclei (ONL). (b) Senataxin (RNA:DNA helicase) staining of mice retinal sections. Lower panel show zoomed view of ONL. (c) RNASEH1 (RNA:DNA ribonuclease) staining of mice retinal sections. Inner segment (IS) of photoreceptor cells (which is rich in mitochondria) show RNaseH1 expression. However, the major expression is observed in INL cells. and ganglion cell (GC) layer.

**Supplementary Figure 2. Genomic instability in absence of Prpf31.** (a) Anti-PRPF31 staining showing depletion of PRPF31 in siRNA-transfected RPE-1 cells. (b) Immunofluorescence was performed using anti- *γH2AX* and *53BP1* antibodies in vasculo-stromal fraction-derived primary cells from *Prpf31 ^+/-^* and *Prpf31 ^+/+^* mice. A representative image of n=2 (for a) and n=2 (for b) independent repeats is presented.

**Supplementary Figure 3. Resistance to irradiation induced cell death observed by terminal deoxynucleotidyl transferase dUTP nick-end labeling (TUNEL) staining in the adult mouse retina.** Post-irradiation, mice were sacrificed at indicated times and retinal sections were used for TUNEL-assay. Untreated or DNAseI treated mice retinal sections were used as negative and positive control for TUNEL assay. The images are representation of n=2 independent repeats, and the same animals as used for Figure 3c.

**Supplementary Figure 4. RNA:DNA hybrids promote the association of ATM with chromatin.** (a) Metamorph-based quantification of nuclear ATM staining by immunofluorescence of pre-permeabilized cells, expressing the hybrid-binding (HB) domain or active RNaseH1. (b) Formaldehyde-based crosslinking of chromatin-associated ATM. Cells treated with H _2_O _2_ in the presence or absence of RNaseH1 were exposed to formaldehyde and lysed in denaturing conditions, and the lysate was analyzed by SDS-PAGE. Loss of signal due to chromatin crosslinking is observed by western blot analysis. The cytoplasmic protein β-actin was used as a control. The representative image of n=4 independent repeats is shown.

**Supplementary Figure 5. Effect of stabilizing RNA:DNA hybrids by hybrid-binding (HB) domain expression on cell cycle**. The HB domain was expressed in RPE-1 (upper panel) and HT1180 cells (lower panel). RPE-1 cells show S-phase accumulation and HT1180 cells show the presence of a sub-G1 population in the presence of the HB domain.

**Supplementary Figure 6. Immunofluorescence analysis of human retinal section.** (a) S9.6 staining to detect RNA:DNA hybrids. The outer nuclear layer (ONL) and inner nuclear layer (INL) are labeled. (b) ATM and 53BP1 expression analyzed by immunofluorescence in human photoreceptor nuclei.

**Supplementary Figure 7. RNA:DNA hybrids stimulate the DNA damage response.** ATM activation and RNA:DNA hybrid formation are interdependent events and important for DNA repair response during oxidative stress. The model shows that RNA:DNA-hybrids (RNA (orange) and template DNA (red)), are sites for ATM binding and activation. We speculate that cells could dissolve RNA:DNA hybrids using enzymes like RNaseH (right panel) or suppress ATM-expression (left panel) to fine-tune ATM-dependent signaling in DNA repair and cell death.
