# Supplementary figures and images for: Formation of 53BP1 foci and ATM activation under oxidative stress is facilitated by RNA:DNA hybrids and loss of ATM-53BP1 expression promotes photoreceptor cell survival in mice
Source: F1000Res. 2018 Aug 10;7:1233. [Version 1] doi: 10.12688/f1000research.15579.1 (PMC6171737; doi:10.12688/f1000research.15579.1)

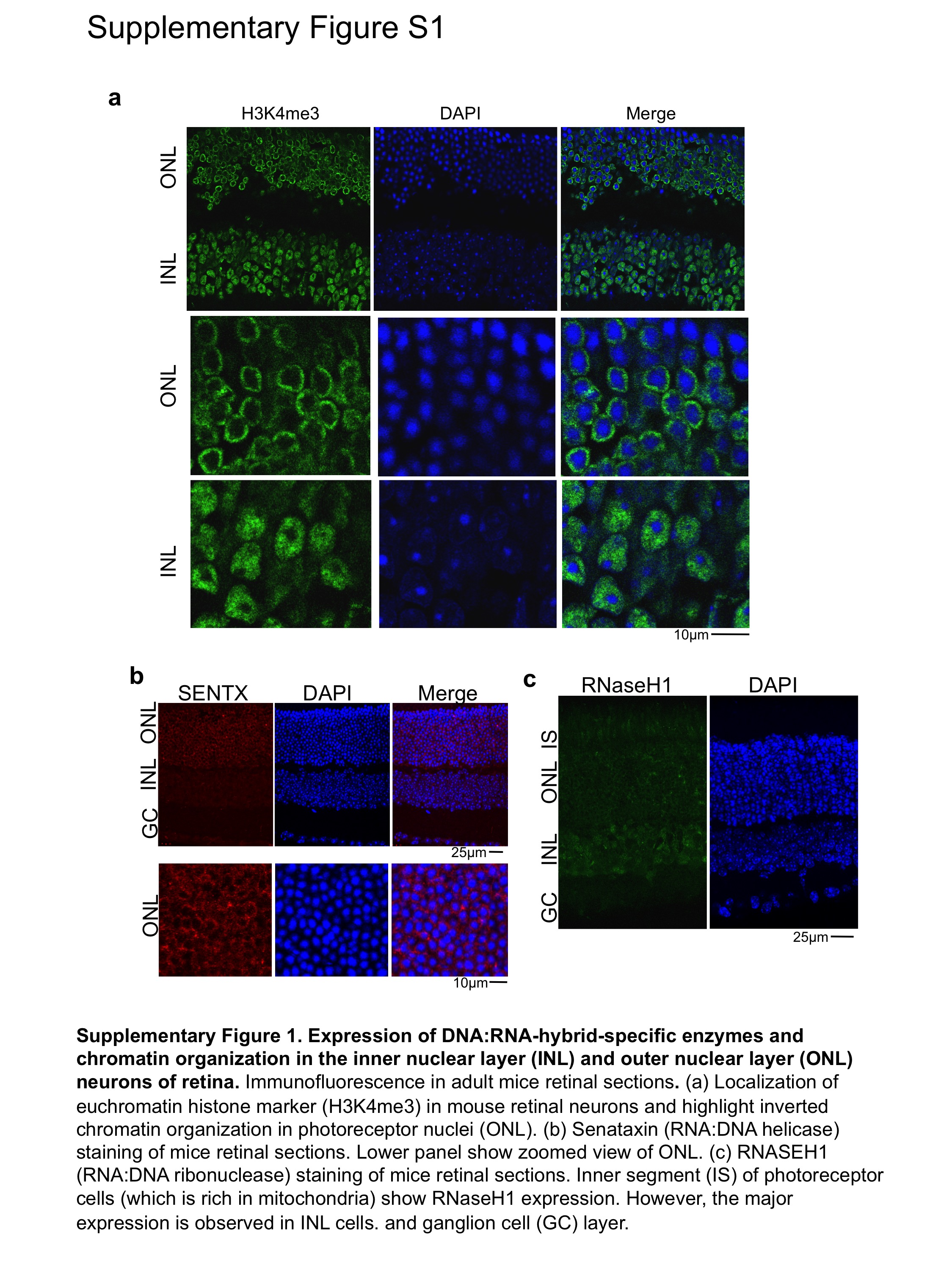

Supplement: Supplementary file 1 [file f1000research-7-16994-s0000.tgz › a6798d59-e51b-40d8-a1bb-3b056ae9864d.jpg]

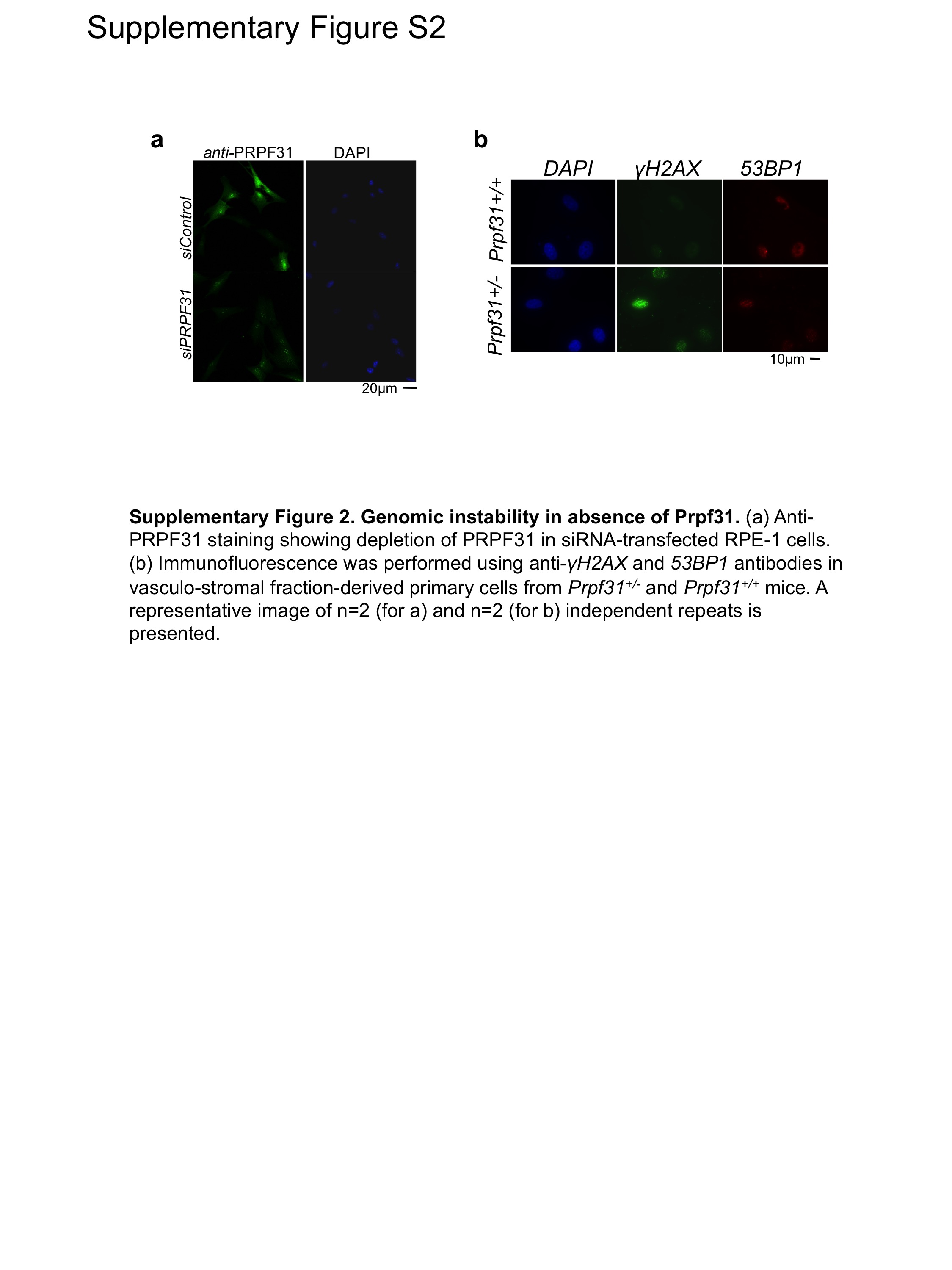

Supplement: Supplementary file 2 [file f1000research-7-16994-s0001.tgz › 124255b0-97c5-4fbd-824f-a90cbd595a54.jpg]

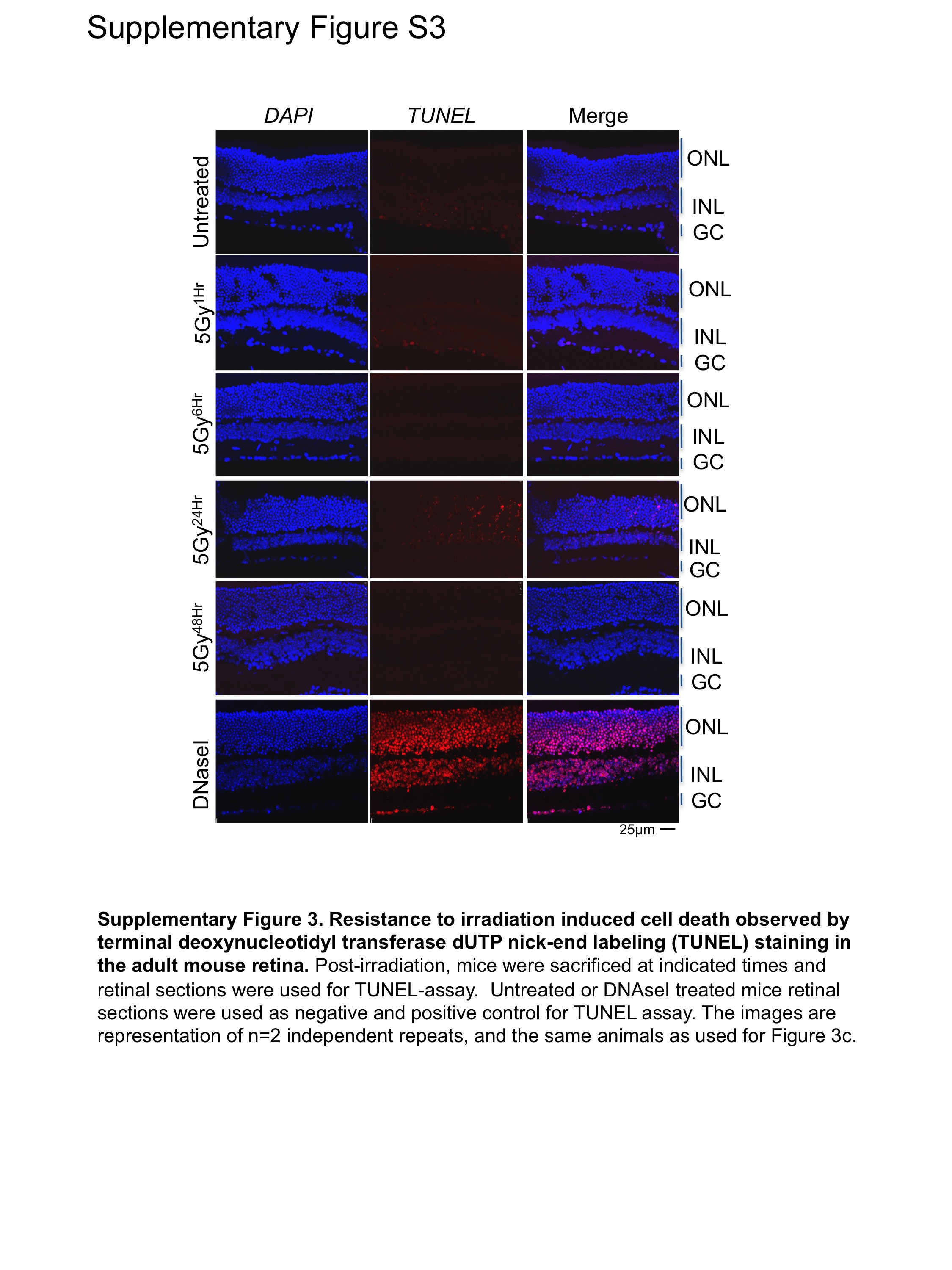

Supplement: Supplementary file 3 [file f1000research-7-16994-s0002.tgz › f1d63324-00c2-4bc7-ba5b-bed99ffe1cf3.jpg]

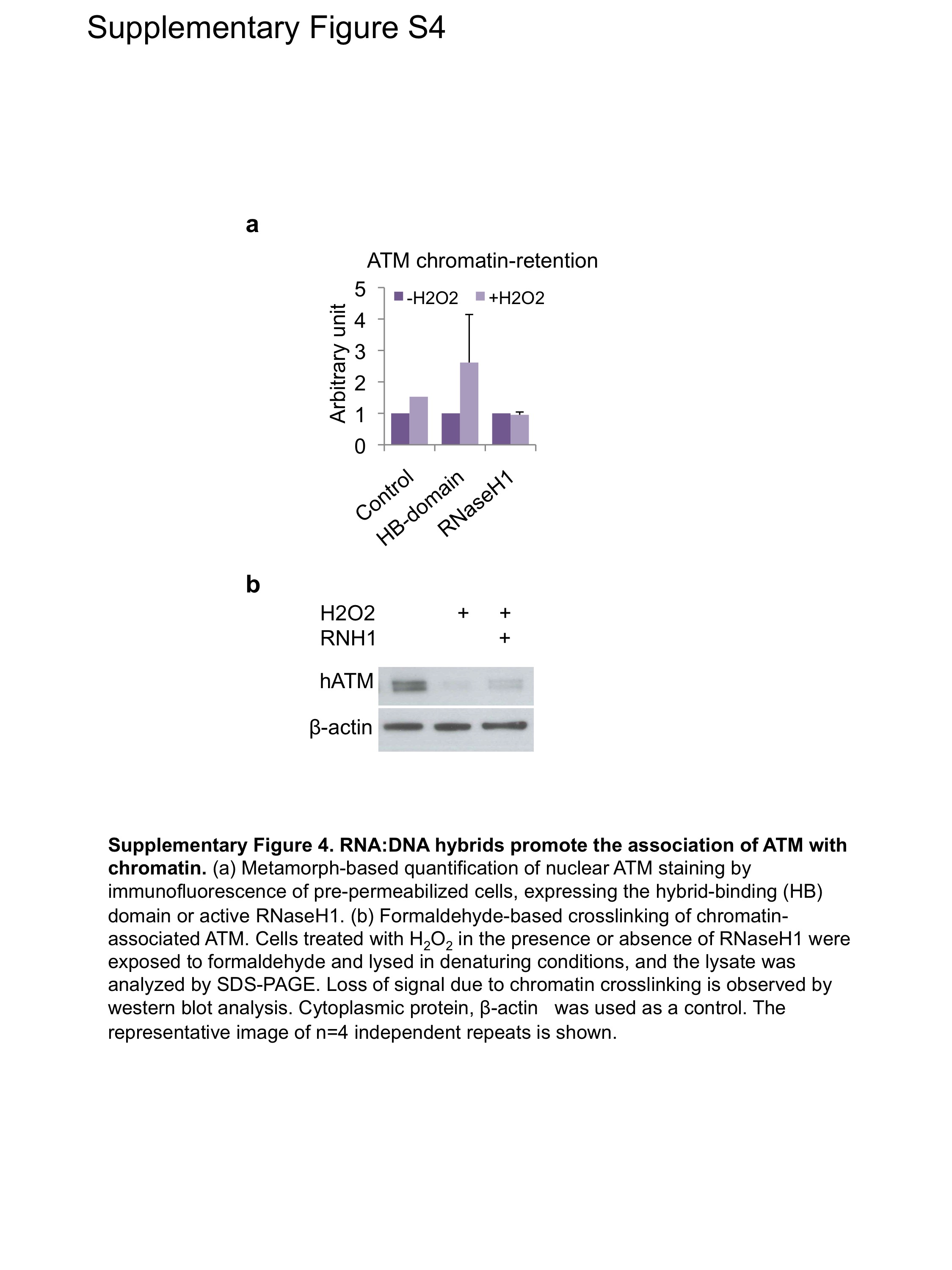

Supplement: Supplementary file 4 [file f1000research-7-16994-s0003.tgz › 18b85809-58fc-4288-88cf-a30b9eb3a91a.jpg]

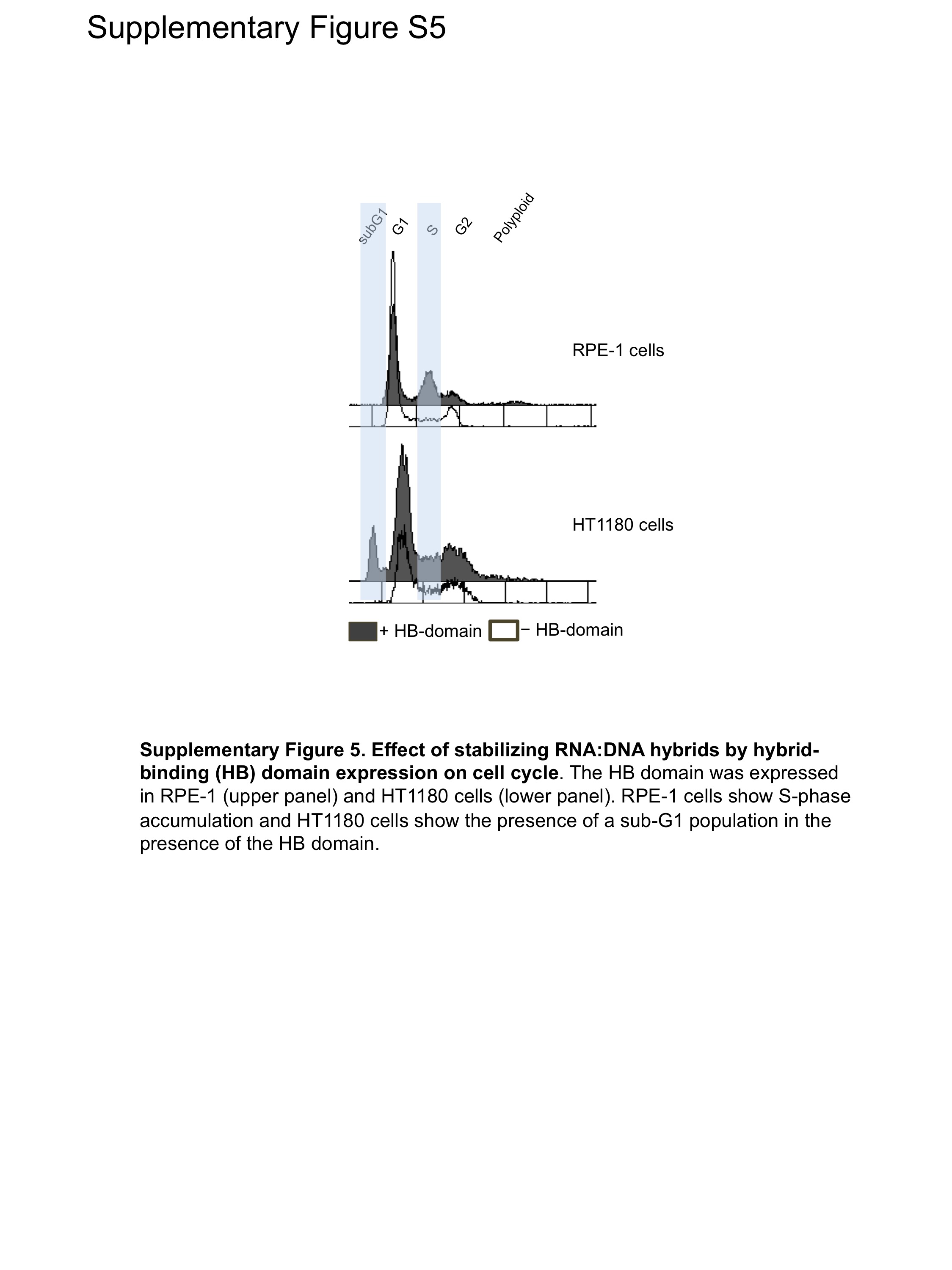

Supplement: Supplementary file 5 [file f1000research-7-16994-s0004.tgz › d74fa6f8-fdbf-4dfb-848a-4c93aa2207a0.jpg]

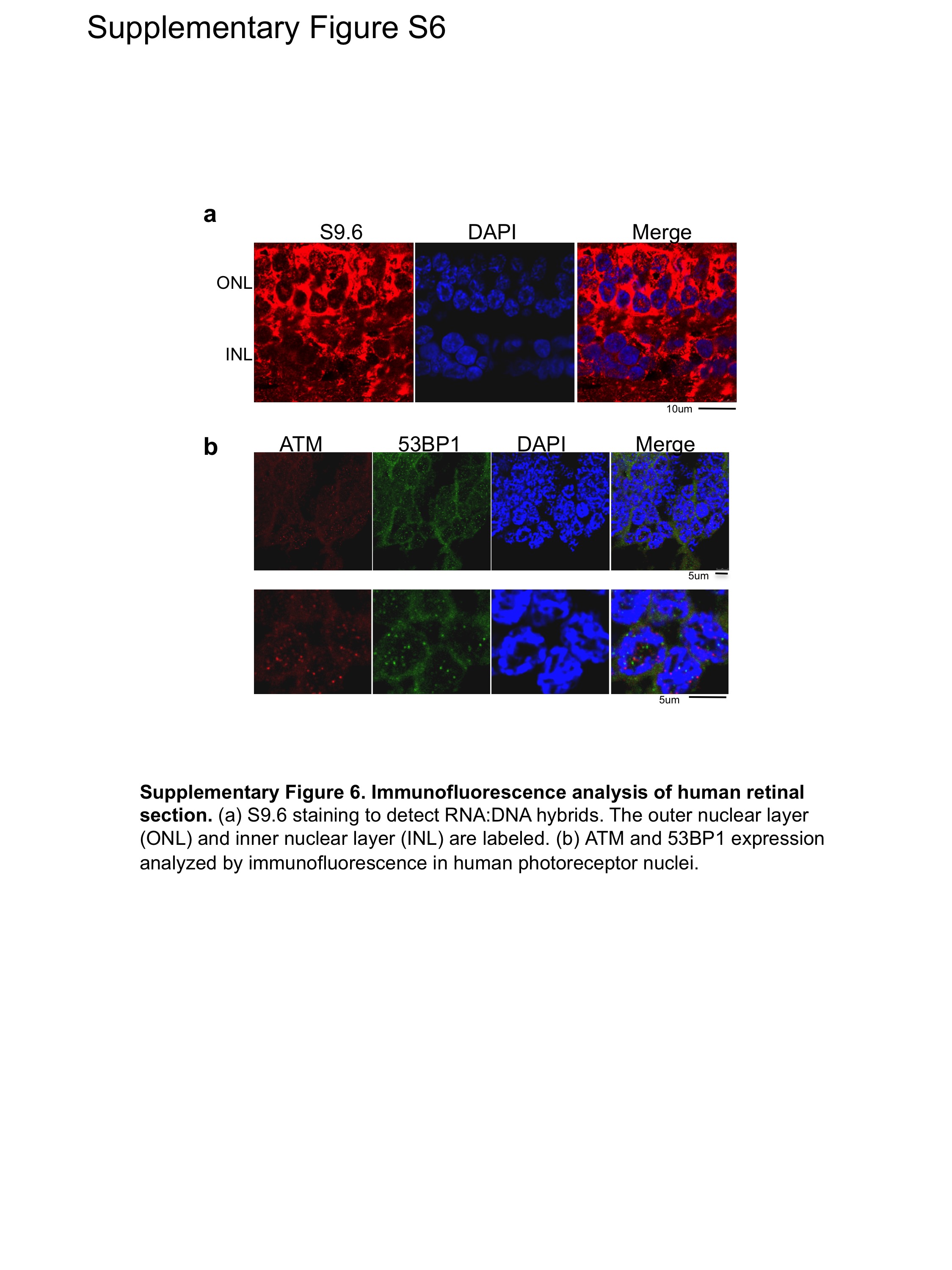

Supplement: Supplementary file 6 [file f1000research-7-16994-s0005.tgz › c0274b0f-1ead-4924-91f6-2eabb0e13cdb.jpg]

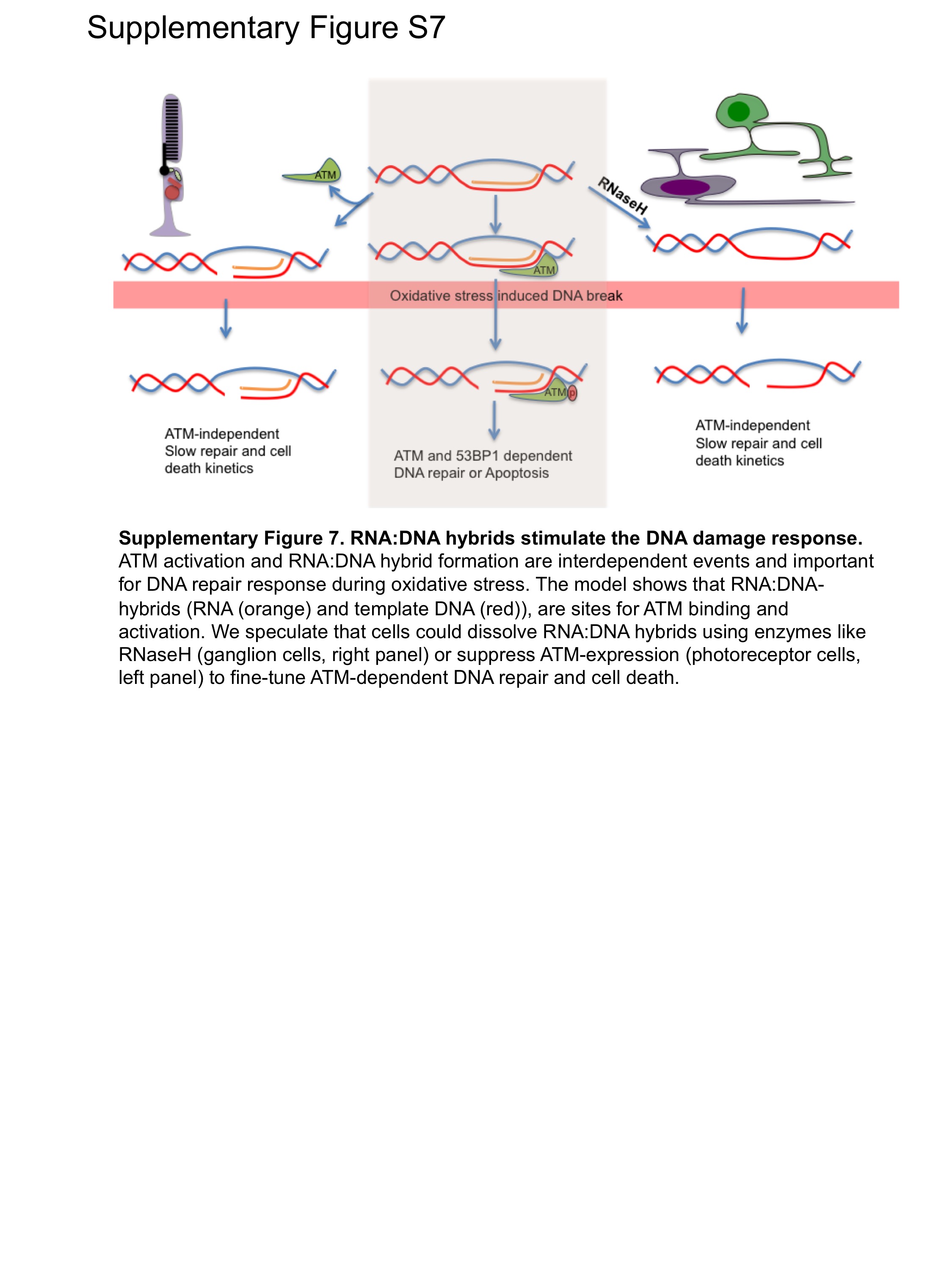

Supplement: Supplementary file 7 [file f1000research-7-16994-s0006.tgz › 1f0af023-9065-4784-81c7-b4bc500b3d46.jpg]
