# Supplementary material for: Formation of 53BP1 foci and ATM activation under oxidative stress is facilitated by RNA:DNA hybrids and loss of ATM-53BP1 expression promotes photoreceptor cell survival in mice
Source: F1000Res. 2018 Aug 10;7:1233. [Version 1] doi: 10.12688/f1000research.15579.1 (PMC6171737; doi:10.12688/f1000research.15579.1)
Supplement: Peer review history

# Peer review: Referee response for version 1

**DOI:** 10.5256/f1000research.16994.r38194

**Published:** 2018-11-05

**Authors:** Maria Tresini

**Affiliations:** 1 Department of Molecular Genetics, Erasmus University Medical Center (Erasmus MC), Rotterdam, The Netherlands

In this manuscript Bhatia et al, provide evidence that suppression of the ATM signaling pathway in murine photoreceptors (PR) is a protective mechanism from oxidative stress/RNA:DNA-hybrid induced apoptosis.

 According to their proposed model, the high levels of oxidative stress PR are naturally subjected to, promote formation of RNA:DNA hybrids which activate ATM and result in p53-mediated apoptosis. Suppression of ATM could therefore have protective effects against retinal degeneration. They support this model by presenting evidence showing that: 1. PRs have higher levels of RNA:DNA hybrids relative to other cells in the retina. 2. Impaired splicing, that has been linked to R-loop formation in other models, can cause RNA:DNA dependent double-strand breaks (DSBs) in retina-derived cell lines, but not in neurons, which they attribute to lack of ATM and 53BP1 expression by the later. 3. In response to ionizing radiation, despite their lower DSB repair capacity, which is independent of ATM, PRs are resistant to apoptosis 4.Oxidative stress in cells activates ATM through an RNA:DNA hybrid dependent mechanism, 5. Absence of ATM delays cell death in a mouse model of retinal degeneration.

 Overall the authors present a large number of interesting data from experiments with mouse retinas and established cell lines (RPE and SVF cells). Their conclusions are consistent with the literature but they also report novel and even controversial findings. The data are well presented and discussed although in some instances a better representation and/or additional controls would strengthen the authors’ arguments. On the somewhat negative side, interesting as the data may be, the large number of issues addressed and experimental models used, result in confusion, loss of coherence and some issues are not addressed or discussed sufficiently. Finally, there are a few statements that should be phrased more carefully as they can be unintentionally misleading.

 Some issues that could be addressed include:

1. The figure supporting the important conclusion that “ *RNA:DNA hybrids specifically accumulate in photoreceptor cells of retina” *should be presented in a manner similar to that in Supplemental figure 1.
2. Can the authors suggest an explanation for the increased RNA:DNA hybrid levels in PRs? Clearly they cannot be formed as a consequence of oxidative stress-activated ATM, as they see a depletion of ATM from ONL.
3. The authors propose that splicing defects (such as silencing or inactivating mutations of PRPF31) induce RNA:DNA-dependent DSBs in cells lines (RPE-1/SVF) but not in retinal neurons. In cultured cells, increased levels of DSBs (assayed here as gH2AX and 53BP1 foci) could be the result of R-loop dependent replication stress and fork collapse. The difference in DSB levels between replicating cells in culture and postmitotic neurons would be more relevant if non-replicating cells (i.e. serum deprived) were used in these experiments or, alternatively, analysis was performed only in G1 cells.
4. Images showing foci formation (e.g. 2a, b, d, e) should be provided at higher resolution as it is impossible to see focal accumulation of gH2AX and 53BP1 in these figures. They should be similar to Fig 3a.
5. The authors report an increase in γH2AX and 53BP1 foci in the retina of postnatal day 20 Prpf31+/A216P mice (text and legend) but the graph actually shows quantification of fluorescence intensity and the γH2AX signal is unchanged. The graph/text should be corrected.
6. The authors use the inactive hybrid-binding (HB) domain of RNaseH1 to stabilize RNA:DNA hybrids. These experiments are central to the paper and RNA:DNA hybrid stabilization should be confirmed by S9.6 immunofluorescence.  
7. The statement that “Noticeably, ATM is shown to promote RNA:DNA hybrid formation on transcribed sites by removal of spliceosomal complex ^32^” is incorrect. While ATM activated by an R-loop mediated pathway can influence spliceosome dynamics and modulate DNA damage-induced alternative splicing, the influence of ATM activity on R-loops was not addressed in this paper.
8. The authors report that oxidative stress activates ATM by a mechanism that depends on RNA:DNA hybrid formation. This is contradictory to the extensive studies of Tanya Paull (e.g. Guo et al, 2010 ^1^) showing direct ATM activation by oxidation. The authors should discuss this. Also, a control showing that H2O2 treatment results in increased RNA:DNA hybrid formation would be useful to strengthen their conclusion.
9. The statement “Clearly, DNA repair activity of ATM and 53BP1 depend on RNA:DNA hybrids” should be rephrased. While this may be true under certain conditions it is too general and can be misleading.

I have read this submission. I believe that I have an appropriate level of expertise to confirm that it is of an acceptable scientific standard, however I have significant reservations, as outlined above.

## References

[1] GuoZDeshpandeRPaullTT: ATM activation in the presence of oxidative stress. *Cell Cycle*.2010;9(24) : 10.4161/cc.9.24.14323 4805-11 10.4161/cc.9.24.14323 21150274PMC3047807

# Peer review: Referee response for version 1

**DOI:** 10.5256/f1000research.16994.r38310

**Published:** 2018-09-27

**Authors:** Florian Frohns

**Affiliations:** 1 Radiation Biology and DNA Repair, Darmstadt University of Technology, Darmstadt, Germany

In this manuscript the authors analyze mechanisms of photoreceptor degeneration. As a major finding they show that photoreceptors show a higher amount of DNA:RNA hybrids when compared to other cells. In several experimental steps the authors then provide evidence that DNA:RNA hybrids can activate ATM. The activation of ATM, in turn, is able to induce degeneration of photoreceptors (PRs) in a mouse model for photoreceptor degeneration. On the other hand the authors provide evidence that ATM is not present in PR cells. Thus, they claim that the accumulation of DNA:RNA hybrids in PRs might be the reason for the compromised DNA damage response, mainly the downregulation of ATM and 53BP1 in this cell type.

 The manuscript is well written and the presentation of the experiments and results is fine. Although the finding on the compromised DNA damage response of photoreceptors has been described earlier, the authors present several new findings that might help to explain these findings in the near future and will significantly contribute to the understanding of DNA:RNA hybrids in the DNA damage response. However, in many cases the authors try to transfer the data from cell lines directly into the retinal tissue which is questionable in several cases. Furthermore, their claim that ATM is not present in PR ignores already published data showing the activity of ATM in this cell type. Thus, in order to improve the work several points have to be addressed:

 1. As presented in Fig. 1, the authors claim that DNA:RNA hybrids are observed in higher levels in photoreceptors. In order to prove this the authors should not only show a representative cross section of a whole retina but also perform quantitative analysis including cells from the INL as well as rod and cone PRs. Furthermore, the authors claim that S9.6 signals in rods are located in peripheral euchromatin of rods. In contrast, in the INL/GCL cell that is also presented in Fig. 1b, S9.6 signals are located in the peripheral heterochromatin. Thus, the authors should comment why in these cells DNA:RNA hybrids would be present in chromatin regions without transcription.

 2. In Fig. 2 the authors present an increasing amount of gH2AX and 53BP1 foci in PRPF31 siRNA-transfected RPE-1 cells and conclude that this increase in DNA damage is due to a higher genomic instability. In contrast, no increased DSBs were found in retinal neurons of PRPF31 deficient mice. The major problem with this finding is that quantification of DNA double strand breaks (DSBs) in the cell lines is not carried out in a cell cycle specific manner. Since the authors did not check the influence of the siRNA treatment on cell cycle distribution and/or progression, these effects might be due to higher amounts of S- or G2-phase cells within one of the samples (since numbers of spontaneous DSBs increase in S-phase due to replication stress and are higher in G2-cells due to the doubled amount of DNA). Thus, the authors should - at least - quantify DSBs specifically for G1-phase cells and exclude S-phase and G2-phase cells from the analysis. The same is true for the  primary cells from *Prpf31 ^+/A216P^* mice. Without such a G1-specific analysis it is not useful to compare the data from a proliferating cell line with strictly postmitotic retinal neurons of adult *Prpf31 ^+/A216P^* mice.

 Another striking finding is the discrepancy between the numbers of gH2AX and 53BP1 foci measured in Fig. 2a and 2b, since many papers have described that the majority of DNA DSBs show both of these markers. The authors should comment on this.

 Finally the authors describe an increased number of gH2AX and 53BP1 foci in PRs of 20 day old *Prpf31 ^+/A216P^* mice when compared with wild type PRs. This is wrong for several reasons: First, the authors present measurements of signal intensities and not the quantification of foci. This should be changed in the text. Second, there is only a significant increase in 53BP1 signal intensity but not in gH2AX. The authors also state that 53BP1 is only expressed in the apical layer of photoreceptor cells. As shown by Müller et al. 2018 (Detection of DNA Double Strand Breaks by γH2AX Does Not Result in 53bp1 Recruitment in Mouse Retinal Tissues) these 53BP1-positive cells are indeed cone photoreceptors. Thus, in order to get a better idea which PR cell type shows this change in 53BP1 levels, the authors should measure 53BP1 levels in both rod and cone PRs independently. Furthermore, high resolution pictures of both cell types should be presented since the actual pictures do not show whether there is indeed focus accumulation or just an increase in pan-nuclear staining.

 3. The authors show that RNAse H1 overexpression has a strong impact on DSB levels. One might wish to see the impact of this overexpression on the level of RNA:DNA hybrids. The authors should present a quantification similar to the one presented in Fig. 7a.

 4. The major message of this manuscript is that the loss of ATM-53BP1 expression promote PR survival. As an evidence for ATM loss the authors present data from western blot analysis of micro-dissected neural retinas in Fig.4. I have several problems with these findings and conclusions: First, on page 11 the authors refer to Donovan et al ^1^ for the procedure of microdissection. After checking this paper one would conclude that the authors have taken the whole retina for this analysis. But in this case these results would not match with the finding that 53BP1 is still present in adult retina (as shown in Fig. 2). Thus, I conclude that microdissection means the isolation of the ONL for western blotting. The authors should clarify their methods. Second, the conclusion that ATM activity is lost seems wrong for the following reason: As Frohns et al ^2^ have shown by the irradiation of Scid mice and additional *in vitro* experiments, cone and rod PRs show a residual ATM activity that is able to phosphorylate gH2AX after DSB induction. Thus, ATM activity should be present in PRs rendering the major message of this manuscript questionable.

 5. The sequence of data presentation in Fig. 5 and 7 is confusing, making it hard to get a clear model of the role of hybrids, ATM expression and its activation in the presence and absence of DNA damage. The authors should try to create a scheme that helps the reader to understand their data. Furthermore, Fig. 5a indicates decreasing gH2AX levels in H2O2 treated and RNAse H1 overexpressing cells when compared to RNAse H1 overexpression alone. In Fig. 7d the opposite can be observed. Why is that?

 6. In Fig. 6a the authors show that H2O2 treatment alone has no impact on the numbers of 53BP1 foci. This is irritating since the same treatment increases gH2AX signals as measured in WB presented in Fig. 5a. Considering the fact that both, gH2AX and 53BP1 are DSB markers the authors should comment on this contradictory finding. In Fig. 6b the authors state that "53BP1 foci consistently co-localize with RNA:DNA hybrids". From 10 53BP1 foci shown in one picture i would score only 5 of them to clearly colocalize with hybrid signals. Thus, the authors should specify that statement or show a quantification.

 7. In Fig. 7d the authors present increasing numbers of gH2AX foci in cells expressing the HB domain. But again the gH2AX analysis is not carried out in a cell cycle specific manner. This is even more of a problem than for Fig. 2 since RPE-1 cells (which likely were used for this analysis, although it is not clearly stated in the legend) show an S-phase accumulation after HB domain overexpression (as shown in Fig. S5). Thus, the increase in gH2AX foci levels might be due to a higher number of S-phase cells (which usually show higher spontaneous DSB levels) in comparison to WT cells.

 8. On page 6 the authors state "Expectedly, ATM-depleted cells are also inefficient in forming 53BP1 foci ( Figure 7a)". I agree that this is expected but still the pictures presented are of too low resolution to underline that statement (no foci visible). Thus, high resolution pictures should be presented.

 9. In Fig. S6b it is shown that ATM and 53BP1 expression is detectable in human PRs. Hereby, the fact that ATM staining shows a punctual or foci-like staining is irritating. If this would be due to its accumulation at DSBs, why is there no colocalization with the also clearly visible 53BP1 foci (have the authors checked for pATM staining)?

 Minor issues:

 On page 4 the authors say that the outer half of the INL is composed mainly of horizontal cell nuclei which is not correct. Horizontal cells are a minority in the outer INL. The most common cell type in this region are bipolar cells. The authors should correct that.

I have read this submission. I believe that I have an appropriate level of expertise to confirm that it is of an acceptable scientific standard, however I have significant reservations, as outlined above.

## References

[1] DonovanSLDyerMA: Preparation and square wave electroporation of retinal explant cultures. *Nat Protoc*.2006;1(6) : 10.1038/nprot.2006.454 2710-8 10.1038/nprot.2006.454 17406527

[2] FrohnsAFrohnsFNaumannSCLayerPGLöbrichM: Inefficient double-strand break repair in murine rod photoreceptors with inverted heterochromatin organization. *Curr Biol*.2014;24(10) : 10.1016/j.cub.2014.03.061 1080-90 10.1016/j.cub.2014.03.061 24794298

# Peer review: Referee response for version 1

**DOI:** 10.5256/f1000research.16994.r37369

**Published:** 2018-09-04

**Authors:** Hemant Khanna

**Affiliations:** 1 Department of Ophthalmology and Visual Sciences, University of Massachusetts Medical School (UMMS), Worcester, MA, USA

Photoreceptors undergo immense oxidative stress throughout the life of an organism. However, it is not clear how these neurons cope with such stress. This manuscript attempts to tackle this question by assessing the role of RNA-DNA hybrid resolution and DNA repair mechanisms during stress. They identify some novel findings, including inefficient DNA repair in photoreceptors and depletion of ATM (a sensor of oxidative stress) levels by P20 in mouse retina. It is also interesting to note that depletion of ATM can have a protective effect in the rd1 mice. These studies also indicate a possible difference between murine and human retinal response to oxidative stress, hence, a different phenotype in mouse models of human RP. The manuscript is well written and sufficiently explained.

 However, there are few minor concerns that should be addressed:

 1.  The ONL staining in Fig 2E may represent cone nuclei. It would be interesting to comment on the possible involvement of cones in this response.

 2. Are the cells used in some experiments primary vascular fraction? If so, were they synchronized as there is a comparison between retinas (post-mitotic neurons) and proliferating cells for oxidative stress responses. It would help to clarify or acknowledge such differences.

I have read this submission. I believe that I have an appropriate level of expertise to confirm that it is of an acceptable scientific standard.

# Peer review: Referee response for version 1

**DOI:** 10.5256/f1000research.16994.r37370

**Published:** 2018-08-30

**Authors:** Travis H. Stracker

**Affiliations:** 1 Institute for Research in Biomedicine (IRB Barcelona) and the Barcelona Institute of Science and Technology, Barcelona, Spain

The manuscript seeks to address the underlying mechanisms of photoreceptor degeneration, particularly that due to ubiquitously expressed proteins. They hypothesize that R-loops may play a role, as several splicing and RNA helicase proteins have been implicated in degeneration and R-loop formation. Using the S9.6 antibody, they find that R-loops are formed in photoreceptor cells of the ONL at higher levels than other cell types. They reason that splicing defects could further increase DNA damage in this high R-loop environment and deplete PRPF31, mutations in which are associated with photoreceptor degeneration. In both RPE1 cells depleted for PRPF31 and primary SVF cells from mice with a PRPF31 knockin mutation, increased levels of gH2AX and 53BP1, markers of DNA breaks, are observed. In the Ki cells, this is suppressed by expression of RNAseH1 that will degrade R-loops, supporting the proposition that the damage is R-loop dependent. However, this signaling was not observed in the ONL layer in vivo but it appears that 53BP1 expression is very low compared to other tissues. They next demonstrate that in response to IR, DNA repair assessed by the appearance and loss of gH2AX is slower and independent of ATM and 53BP1, both of which appear to be lowly expressed in the mature retina. Interestingly, they identify a truncated form of ATM mRNA expressed in the retinal cells that may lead to a null or kinase dead ATM protein. Next they show that peroxide activates ATM in a manner dependent on RNAseH1 (thus, presumably R-loops) but in contrast, 53BP1 foci, that co-localize with R-loops are suppressed by peroxide. Analysis of ATM deficient cells using the HB domain to stabilize R-loops revealed fewer R-loops and 53BP1 foci, suggesting that ATM promotes R-loops formation to recruit 53BP1. Unexpectedly, gH2AX signal is strongly increased in the presence of peroxide and RNAseH1 expression, potentially reflecting deficient repair. Finally, the authors show that ATM loss promotes photoreceptor survival and ONL thickness in a mouse model.

 The manuscript is overall well written and the data clearly presented. There are a number of new and interesting findings regarding the expression of damage factors and their influence on the retina. However, the logic and flow of the manuscript are somewhat confusing in places. The authors switch between post-mitotic tissues and proliferating cells and there is some apparently conflicting data and overstatements that could use clarification. Here are some specific comments and suggestions to address these points.

1. Under the header “RNA:DNA hybrids specifically accumulate in photoreceptor cells of retina” the authors state: “Higher levels of RNA:DNAhybrids are observed in adult photoreceptor nuclei than in the other retinal neurons”. Based on the data provided, this claim, that is central to the manuscript, is not well substantiated. Essentially a single cell representing multiple cell types is shown in Figure 1b in comparison to the ONL cells. To support this definitive statement that *R-loops are specifically higher in the ONL*, a quantitative or even semi-quantitative analysis (for example, nuclear intensity in each population) is needed. At a minimum, additional examples of other cell types should be provided to demonstrate some level of specificity.
2. Could the discrepancy in gH2AX and 53BP1 signaling between Ki cells in culture and in vivo be due to the proliferative status of the cells? Are the ONL cells analyzed all post-mitotic? Additions to the text or a Ki67 stain of the retinal layers could address this issue, as DNA replication and mitosis could exacerbate the levels of damage.
3. In Figure 5, the authors show that H202 activates ATM and that this is suppressed by RNAseH1 expression, thus implicating R-loops (very interesting). However, it is not directly shown that H202 actually increases R-loops. This should be stated and referenced if already known or shown directly.
4. The authors state: “Clearly, DNA repair activity of ATM and 53BP1 depend on RNA:DNA hybrids.” I do not agree with this unqualified statement, particularly as their repair activity in relation to these hybrids is not measured in this work. Previous work has implicated R-loops in ATM activation (Tresini et al, Nature 2015) or proposed that R-loops impair ATM-mediated repair (Walker et al, Nature Neuroscience 2017) while the authors here implicate ATM activity in R-loop formation. The cause-effect relationship of ATM, R-loops and repair thus remains somewhat unclear to me. This could be clarified by a more substantial discussion or model to clarify the position of the authors.
5. The observation in Figure 7d that RNAseH1 increases gH2AX dramatically is very interesting but could use clarification, in part because this result is not apparent in Figure 5a, where essentially the same experiment is done if I understand correctly. In 7d, gH2AX levels are reduced by H2O2+RNAseH1 expression in contrast to 5a where they are close to doubled. Considering the results of 5a show reduced ATM activation in the peroxide+RNAseH1 setting, the increase in gH2AX observed in 7d must therefore be almost completely ATM-independent. The results of Figure 4b following IR also show ATM-independent activation of gH2AX following IR. The model that ATM induces R-loops to recruit 53BP1 for repair is therefore not fully supported. Again, this could be clarified via the text or a model.
6. The manuscript starts by focusing on the ONL in Figure 1 and pointing out that it has high R-loops and in Figure 4 showing it has low ATM/53BP1. Therefore, the fact that ATM loss protects the tissue from R-loops (Figure 8) is somewhat counterintuitive if ATM is not there to respond to R-loops or to promote their formation. This would suggest that ATM-dependent tissue loss would occur prior to postnatal day 10 when ATM is expressed. If R-loops are indeed ATM dependent, as shown in Figure 7, it would predict that the high R-loops in the ONL must occur before postnatal day 10. Have R-loop levels been assessed at different time points? 
7. The title implicates 53BP1 in photoreceptor survival while this is not actually demonstrated. ATM dependent activation of p53 is independent of 53BP1 and a pro-apoptotic role of ATM in this setting could therefore also be independent of 53BP1.

Minor issues

1. In Figure 3 legend the caption of (a) states …..after exposure to 5 Gyrase.. rather than 5 Gray.
2. No size markers are shown on western blots, these should be added for reference and reproducibility.
3. The Figure 5, 6 legends make no reference to the cell or tissue type being used for the experiments.
4. Given that ATM and 53BP1 expression are lost in the retina following postnatal day 10, it becomes very relevant the age of the tissues assayed in Figure 2, 3 and 8. This should be stated in the legends.

I have read this submission. I believe that I have an appropriate level of expertise to confirm that it is of an acceptable scientific standard, however I have significant reservations, as outlined above.
